# Gridded emissions and land-use data for 2005–2100 under diverse socioeconomic and climate mitigation scenarios

**DOI:** 10.1038/sdata.2018.210

**Published:** 2018-10-16

**Authors:** Shinichiro Fujimori, Tomoko Hasegawa, Akihiko Ito, Kiyoshi Takahashi, Toshihiko Masui

**Affiliations:** 1 Department of Environmental Engineering, Kyoto University; C1-3-361, Katsura-Campus, Nishikyo-ku, Kyoto-city, 615-8540, Japan; 2 Center for Social and Environmental Systems Research, National Institute for Environmental Studies (NIES), 16-2 Onogawa, Tsukuba, 305-8501, Japan

**Keywords:** Climate and Earth system modelling, Climate-change impacts, Climate-change mitigation

## Abstract

Information on global future gridded emissions and land-use scenarios is critical for many climate and global environmental modelling studies. Here, we generated such data using an integrated assessment model (IAM) and have made the data publicly available. Although the Coupled Model Inter-comparison Project Phase 6 (CMIP6) offers similar data, our dataset has two advantages. First, the data cover a full range and combinations of socioeconomic and climate mitigation levels, which are considered as a range of plausible futures in the climate research community. Second, we provide this dataset based on a single integrated assessment modelling framework that enables a focus on purely socioeconomic factors or climate mitigation levels, which is unavailable in CMIP6 data, since it incorporates the outcomes of each IAM scenario. We compared our data with existing gridded data to identify the characteristics of the dataset and found both agreements and disagreements. This dataset can contribute to global environmental modelling efforts, in particular for researchers who want to investigate socioeconomic and climate factors independently.

## Background & Summary

There are three main domains of future climate change research: climate simulations performed mainly with earth system models (ESMs) or general circulation models using greenhouse gas (GHGs) and air pollutant (AP) emission data and land-use data; impact, adaptation, and vulnerability (IAV) models that assess climate impacts using future climate information and socioeconomic conditions; and GHG and AP emission scenarios and climate change mitigation policy assessments performed with integrated assessment models (IAMs). However, gridded emissions and land-use data are necessary for both ESM projections and IAV assessments.

Over the last ten years, the IAM community has developed multisets of scenarios for use by other climate research communities. The first set of scenarios includes the Representative Concentration Pathways (RCPs)^1^, which have been used extensively in studies cited by the Fifth Assessment Report of Intergovernmental Panel for Climate Change (IPCC) and conducted mainly by the ESM and IAV communities. The RCPs include main climate forcing emissions, atmospheric concentrations, and land-use data. Carbon dioxide (CO_2_) is provided as regionally aggregated total emissions, while nitrous oxide (N_2_O), methane (CH_4_), and other AP species are provided as gridded data. In addition, land-use information is provided as gridded data. The RCPs include four global warming levels represented by radiative forcing at stabilised points: 2.6, 4.5, 6.0, and 8.5 Wm^−2^.

The Shared Socioeconomic Pathways (SSPs) represent another set of scenarios that cover a wide range of plausible socioeconomic development trends over the century^2^. The scenarios compile narratives^3^ that describe the main characteristics of human and societal development patterns and quantitative information, which consider population, gross domestic product (GDP), energy, emissions, and land use, among others^4^. The SSPs include five sets of scenarios, namely, SSP1 to SSP5^5–9^.

The SSPs employ a concept called scenario matrix architecture, which has a two-dimensional space comprising socioeconomic patterns and climate mitigation levels. The former is classified by the SSPs and the latter is represented by radiative forcing levels, which are sometimes interpreted as RCPs, although they differ from the RCPs because 1.9 and 3.4 Wm^−2^ forcing levels are added to the original RCPs and, more importantly, scenarios that achieve the same levels of mitigation are not identical across the SSPs (e.g. the 2.6 Wm^−2^ mitigation scenario under SSP1 differs from that under SSP2).

Similar to the RCPs, the SSPs are supposed to be for use by the ESM, IAV, and IAM communities; however, the number of scenarios in the full scenario matrix is too large for use in ESMs. As a compromise, the ScenarioMIP (Scenario Model Intercomparison Project) of the CMIP6 (Coupled Model Intercomparison Project Phase 6) created a protocol to select representative scenarios for use in CMIP6 exercises that allows them to cover sufficiently a rational and practical range of mitigation levels and socioeconomic development patterns^10^. Ultimately, the IAM community is expected to provide gridded emissions and land-use information for the aforementioned selected scenarios (this dataset is referred to as ScenarioMIP data hereafter).

Although ScenarioMIP data could be sufficient for CMIP6 and IAV analysis which uses CMIP6 outcomes for many purposes, there may be additional needs for gridded data. For example, the current ScenarioMIP protocol could lack combinations in the SSP/RCP matrix that might be of interest to certain IAV or ESM groups. In addition, the ScenarioMIP data were originally created with five IAMs with different characteristics in the representation of emissions and land-use dynamics. This means that these data encompass inherent SSP characteristics, but also IAM model fingerprints, which do not allow users to isolate pure SSP dimensions.

To address these issues, we created gridded emissions and land-use data under a full SSP/RCP matrix generated by a single IAM, the Asian-Pacific Integrated Model (AIM), which we call the AIM-SSP/RCP. The data cover 2005–2100 and complement the ScenarioMIP data. The main goal of this data publication is to fill the gap between currently publicly available data and the demand for datasets to enhance climate research under consistent scenario assumptions, and can be used in the broader context of scenario-based environmental research (e.g. biodiversity research^11^).

To this, although we applied similar methodology to that described in Fujimori *et al.*^12^ and Hasegawa *et al.*^13^ for emissions and land-use respectively, our paper has different goals from these two studies, which derived methodological implications of climate simulation outcomes and land-use model integrations, respectively. The aims of this paper are as follows. First, we provide all combinations of SSPs and RCPs and its description while these papers just examined one of the examples. Second, this paper explains how our data is different from ScenarioMIP data from the point of view of advantages and disadvantages of our AIM-SSP/RCP data compared to ScenarioMIP data. Third, data file naming convention and how it can be used, and technical data comparison with the currently available data is shown.

## Methods

### Overall framework

**Figure 1:**
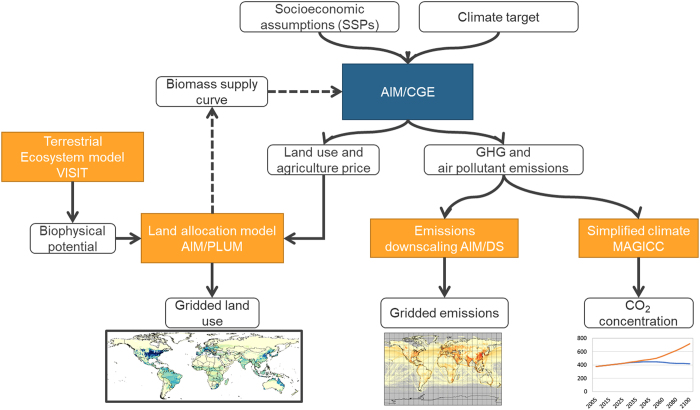
Overview of the calculation method and flow used to create the datasets. Biomass supply curve feedback was not used in this study, although it is usually activated in the AIM simulation. Items marked by dashed line are generally available but were not activated for this particular study.

Figure 1 illustrates the method used to calculate the AIM-SSP/RCP gridded emission and land-use data. First, an AIM/computable general equilibrium (CGE) model representing all economic activities was used to compute energy, emissions, and land-use information. The AIM/CGE is a global model that classifies the world into 17 aggregated regions (Table 1). Then, an AIM/PLUM (integration Platform for Land-Use and environmental Modelling) disaggregated regionally aggregated land-use data into a gridded basis using land biophysical productivity potential, water, and land conservation information as inputs. Meanwhile, emissions were downscaled using an AIM/DS (DownScaling) model. Since some climate models require CO_2_ concentrations for their simulation, we prepared a CO_2_ concentration dataset, albeit not on a gridded basis, using the simplified climate model MAGICC (Model for the Assessment of Greenhouse-gas Induced Climate Change) version 6^14^.

We generated 24 scenarios, as outlined in Table 2. The baseline case does not include a climate mitigation policy. The climate conditions are represented by four RCP levels (2.6, 4.5, 6.0, and 8.5 Wm^−2^) and three additional forcing levels (1.9, 3.4, and 7.0 Wm^−2^). For comparison, the case with a forcing level of 7.0 Wm^−2^ roughly corresponds to the SSP2 and SSP3 baseline cases. The levels of 1.9 and 3.4 Wm^−2^ are policy-relevant in the sense that these mitigation levels are used in the 1.5 and 2 °C scenario sets^15^.

### AIM/CGE

#### Model overview

The AIM/CGE model is a recursive-dynamic general equilibrium model that covers all regions worldwide. Details of the model structure and mathematical formulas are provided by Fujimori *et al.*^16,17^. The main inputs of the AIM/CGE are population, GDP, food preferences, assumptions of energy technology progress on both the supply and demand sides, and air pollution control level. The model provides energy consumption, agricultural and land-use indicators, and GHG and AP emissions. The AIM/CGE considers CO_2_, CH_4_, N_2_O, and fluorine gas to be GHGs, while black carbon (BC), carbon monoxide (CO), ammonia (NH_3_), non-methane volatile organic compounds (VOCs), mono-nitrogen oxides (NO_X_), organic carbon (OC), and sulphur oxides (SO_X_) are treated as APs. The regional, geographical, and industrial classifications are shown in Table 1, Supplementary Figure S1, and Supplementary Table S1, respectively.

With respect to land-use and agricultural representation, the AIM/CGE model employs a land-nesting strategy, similar to the approach used in previous studies^18^. In this approach, land is categorised in one of three agro-ecological zones (AEZ) that are aggregated from the original 18 classifications, where there is a land market for each zone. The allocation of land by sector is formulated as a multinomial logit function to reflect differences in substitutability across land categories with land rent. As such, the function assumes that landowners in each region and AEZ decide on land sharing among options with land rent depending on the production of each land unit (i.e. crops, livestock, and wood products). One approach to using nesting strategies is based on the assumption that land regions are small enough that all competing options are equally substitutable. This assumption implies that it is as easy to switch from forest to wheat as it is to switch from corn to wheat; however, in reality this conversion would not occur unless wheat was more profitable than forest or corn. To calibrate the functions for both managed and unmanaged land in the base year, because no data were available for unmanaged land, we assigned the average base-year land rent of managed land to unmanaged land. It should be noted that the land rent of forest includes both revenue from wood products and the carbon stock price, as evaluated in the case of a particular climate mitigation scenario.

#### Base year data inputs

CGE models use a social accounting matrix (SAM) to calibrate the model parameters. To more precisely and realistically assess the energy flow and GHG emissions, CGE models should account not only for the original SAM, but also for energy statistics. The Global Trade Analysis Project (GTAP)^19^ and energy balance tables^20^ were used as the basis for the SAM and energy balance table, and data were reconciled with other international statistics, such as national account statistics^21^. The concept behind the reconciliation method has been described previously^22^. GHG and air-pollutant emissions were calibrated to EDGAR4.2^23^. For the land-use and agriculture sectors, agricultural statistics^24^, land-use RCP data^25^, and GTAP data^26^ were used as physical data. The agricultural quantity consumption was converted into caloric intake using a conversion factor derived from agricultural statistics^24^.

### AIM/PLUM

#### Model overview

The AIM/PLUM is a global land-use allocation model used to downscale regionally aggregated land-use projections into a spatially gridded land-use pattern for the interactive assessment of human activities and biophysical elements^13^. In this study, the regionally scaled land demand provided by the AIM/CGE model (17 regions) was spatially distributed into grid cells (0.5° × 0.5°). The land allocation was based on economic efficiency (profit maximisation), where a landowner was assumed to decide the mix of land uses to obtain the highest profit for a given biophysical land productivity (production per unit area). Because land was allocated for each region with the same regional classification as that used in the AIM/CGE, land transactions across regions were considered in the AIM/CGE but not in the AIM/PLUM. Seven crop types, with or without irrigation, were considered. Table 3 lists the land classifications considered in the AIM/PLUM. See Hasegawa *et al.*^13^ for details and these methods are summary versions of descriptions in our related work^13^.

#### AIM/PLUM input data.

##### Profit and cost information

Profit was represented as the revenue minus cost. Revenue from production was the product of the commodity price multiplied by the unit area production. Revenue from afforestation included revenue from both the carbon sequestration of the forest and benefits of forest restoration. Cost information was provided by the AIM/CGE model and was uniformly applied to the grids in each region. The costs of crop production and afforestation were calculated as the total sum of capital, labour, and intermediate costs divided by the land area for each sector. Land conversion costs included road construction costs, irrigation costs, and payments for land-use emissions. Road construction costs were calculated from road length (https://worldroadstatistics.org/) and the road construction cost per unit. Regional irrigation cost was based on the work of Nelson *et al.*^27^ and applied to different regions after adjusting according to income level.

##### Land productivity and carbon stock density

For the crop yields of irrigated or rain-fed areas, we used the mean yields from 1990 to 2004 calculated with the Lund-Potsdam-Jena managed Land Dynamic Global Vegetation and Water Balance Model (LPJmL)^28^. The mean yield is weighted by this yield information. All crop yields considered CO_2_ fertilisation. The yields of 13 crops from the LPJmL were aggregated into seven crop classifications in the model in each grid using the crop production of the country in which the grid was situated^24^ as the weight.

Carbon stock density was based on estimates from the Vegetation Integrative SImulator for Trace Gases (VISIT)^29^, while energy-crop yield was derived from the hydrological model H08^30^. For forest carbon sequestration, we referred to the carbon stocks of the AEZs^31^, which were allocated to the grids according to the AEZ of each grid. The potential carbon sink of afforestation and changes in annual biomass growth along with forest age were calculated using a timber yield function^32^ with parameters estimated assuming a saturated IPCC carbon stock level.

##### Reference land-use map

As a reference land-use map for the base-year allocation, cropland maps were used by aggregating 175 types into the crop classifications of the model^33^. RCPs^25^ were used for settlement, ice, or inland water while UNEP-WCMC (www.protectedplanet.net) was used for protected land. The irrigation ratio in the base year was calculated by multiplying the gridded map of the current irrigation ratio (MIRCA2000) and cropland ratio by the crops^33^. When there were inconsistencies in the land area among different sources, land types other than cropland and pasture were adjusted. Settlement, ice or water, and protected area were fixed for the entire period.

### AIM/DS

#### Model overview

The AIM/DS is a model used to downscale regionally aggregated emissions based on an AIM/CGE model on a grid-based map with 0.5° resolution. The algorithm is differentiated by sectors, and the emission sources are assigned to one of three groups (Table 4). In group 1, GDP and population are the drivers of emissions. This algorithm is adopted mainly for energy-related emissions, which we assumed to have a relationship with GDP or population. Group 2 is downscaled in proportion to the total regional emissions. The base year map information is scaled up or down according to the total regional emissions. The base year represents the starting year of the AIM/CGE simulation (i.e. 2005). Therefore, the spatial distribution pattern for future scenarios is the same as that of the base year. Group 3 is downscaled in proportion to the total global emissions of the base year spatial map. The basic logic in the case of group 3 is the same as that of group 2, but applied to cross-border sectors.

We used national-level population, urbanisation rate, and GDP data to generate the spatially gridded populations and GDPs. The base consisted of 2.5′ data from the Gridded Population of the World^34^. As the initial values, we used 0.5′ population data to produce population distributions within the 2.5′ grid cells. Initial population data and national urban population data were used in urban areas. We set population density thresholds based on the initial populations in the 0.5′ grids so as to match national urban populations. We treated the 0.5′ grid cells above the threshold as urban cells and those below the threshold as rural cells. For the 30′ grid cells, we used urban population/area ratios as the urban index. The data were sourced from the Greenhouse Gas Initiative database provided by the International Institute for Applied Systems Analysis^35^.

We used the rank-size rule to estimate the populations of the urban grid cells, which is an empirical law used to estimate previous city populations^36^ but is also applicable to future populations. Then, the GDP distributions were allocated based on the populations considering several geographical constraints, including mountains, waterbodies, and urban sprawl. These methods are summary versions of descriptions in our related work^12^.

### Code availability

The code and relevant configuration files are in the data repository (Data Citation 1) as “AIMGridemissioncode.zip” and “AIMGridLandusecode.egg” for emissions and land-use respectively. After unzip, there are batch files to make emissions and land use data. For the emissions, /AIMGridemissioncode/shell/python.bat is the batch file that make all emissions NetCDF files. To run the model, python and relevant libraries, and GAMS need to be installed and moreover, both software paths also need to be configured. Regarding land-use, there is a file “AIMGridLandusecode.egg/prog/shell/csv2nc.bat” which processes the NetCDF file. The execution is confirmed under the Cygwin and NetCDF relevant packages installed environment.

## Data Records

The data repository, including gridded emission, land-use, and CO_2_ concentration data, is stored on Harvard Dataverse (Data Citation 1) and the AIM modelling team website (Data Citation 2). For the latter database, the data are available in two locations (http://www.nies.go.jp/doi/10.18959/20180403.001-e.html, http://www-iam.nies.go.jp/aim/data_tools/aimssp/aimssp.html ). The file format for the gridded file is NetCDF version 4, which is used widely in the climate research community. The gridded emissions and land-use data are represented in 0.5° × 0.5° grids, totalling 259,200 pixels globally.

The data from the emissions downscaling are stored for each scenario and species. The file names consist of three components:

1. Socioeconomic assumption
2. Climate mitigation level
3. Species

The naming convention for each file is AIM-SSPRCP-Emissions_$[socioeconomic assumption]_$[climate mitigation level]_$[species]_$[version]. The elements in each of the three dimensions are listed in Table 5. For example, the BC emissions in SSP1 under a 2.6 Wm^−2^ climate mitigation level are stored in the file named “SSP1-26-BCE.nc”. Moreover, emissions are differentiated by source sector, as shown in Table 4, which are named as listed in Table 6. All data are represented as the mean (kg/m^2^/s). Zero values are represented as “NaN”. The full list of file names and their corresponding species and scenarios are shown in Supplementary Table S2.

The land-use data from the land-use downscaling are stored for each scenario (all land-use categories in Table 3 are in the same NetCDF files). Supplementary Table S3 presents the full list of files. The naming convention for each file is AIM-SSPRCP-LUmap_$[socioeconomic assumption]_$[climate mitigation level]_$[version]. The units are the fractions of each land-use category in each pixel. For example, if the cropland share of a grid is 60%, the value in that cell is 0.6.

The CO_2_ concentration data are stored in a single csv file and include two dimensions, scenario and year.

## Technical Validation

We compared our downscaled emissions and land-use data with available data for the historical period overlapping with the AIM-SSP/RCP coverage (2005 and 2010) for use in CMIP6. The emissions data included CEDS (Community Emissions Data System)^37^ and land-use data included LUH2 (Land-use Harmonisation)^38^.

**Figure 2:**
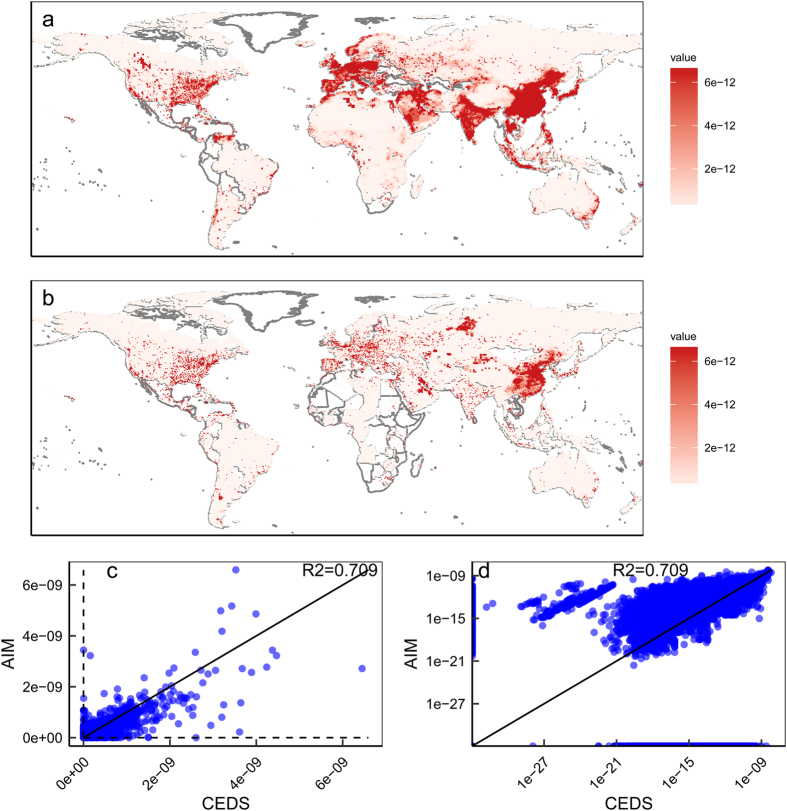
Comparison of downscaled SO_2_ emissions in the base year 2005. (**a**) Spatial emission density for AIM-SSP/RCP. (**b**) Spatial emission density for CEDS. (**c**) the datasets on normal scales. (**d**) The datasets on logarithmic scales. All panels use the same unit (kg/m^2^/s).

We used the SO_2_ emitted by the energy sector in 2005 as an illustrative example of emissions. The two datasets showed certain agreements; however, the coefficient of determination (R^2^) was not very high, and there were some spatial discrepancies in emissions (Fig. 2a and b). For example, the AIM-SSP/RCP data were spread thinly and broadly (e.g. in China), whereas the CEDS was condensed in limited places. In addition, many points had a value of zero for one dataset but a non-zero value for the other (Fig. 2c). There are several possible reasons for this. First, the total emissions for each country or region differ between the scenarios and CEDS. In addition, our data were calibrated to EDGAR4.2, which differs from CEDS. Similarly, the 2005 gridded data were calibrated to the RCP data in the AIM-SSP/RCP, which could differ from CEDS. In principle, there are no observations for global AP emissions; therefore, we could only compare the datasets, but could not judge which was better.

The results of the comparisons of 2010 and NO_X_ are presented in the Supplementary File 1, which showed similar tendencies (Supplementary Figure S2–Supplementary Figure S4). The sectors [e.g. transport and building (residential and commercial)] presented different pictures (Supplementary Figure S5 and Supplementary Figure S6), with large discrepancies in absolute terms and modest discrepancies on a logarithmic scale. Overall, the R^2^ values were low, likely due to discrepancies between the base data (CEDS and EDGAR), of which the reliability cannot currently be easily assessed.

To explore the differences between these two datasets more systematically, we present two relevant types of analysis for the base year data. The first is the level of agreement between the datasets for the 17 aggregated regions that are used for classification in AIM/CGE (Table 7). There are global variations across sectors and species but the ratios of AIM-SSP/RCP to CEDS values are in the range 0.66–1.11, which can be interpreted as modest differences. Individual regions could exhibit more variations. The energy sector, which has a large share of both species, shows relatively small differences while the building sector, which is a small contributor to the total emissions, exhibits high discrepancies.

Secondly, we performed a regression analysis according to the following equation:

$$\left|\log10\left(\frac{{\mathrm{AIM}}_{i}}{{\mathrm{CEDS}}_{i}}\right)\right|=\alpha_{r}+C+\varepsilon_{i},$$

where AIM_i_ and CEDS_i_ are the emissions of AIM-SSP/RCP and CEDS in grid cell *i*, *a*_*r*_ is regional bias parameter, *c* is intercept and *ε* is an error term. The estimates of these parameters are shown in Table 7. This analysis produces different results from those described above. The energy sector has a high discrepancy, which can be seen in the high intercept value for SO_2_. This implies that there are numerous grid cells with high discrepancies, which can also be inferred from Fig. 2. On the other hand, building, industry, and transport exhibit small differences in general. Regionally, we could not find a systematic discrepancy (Table 8).

Since it is worthwhile for users to know how the ScenarioMIP and AIM-SSP/RCP data are different, we compared these two datasets for future scenarios. In ScenarioMIP, SSP3-baseline (SSP3-radiative forcing 7.0 W/m^2^) scenarios are comparable because AIM provides this scenario. As examples, SO2 and NOx, and the years 2015 (ScenarioMIP starting year), 2050 and 2090 are selected. We found that the differences are similar over time, which means that the base year has strong influences on the future gridded emissions as well (Supplementary Figure S7-Supplementary Figure S12). One of the reasons why the base year fingerprint remains strong in the future is the characteristics of the SSP3-baseline scenario, in that this scenario does not include strong policy intervention.

**Figure 3:**
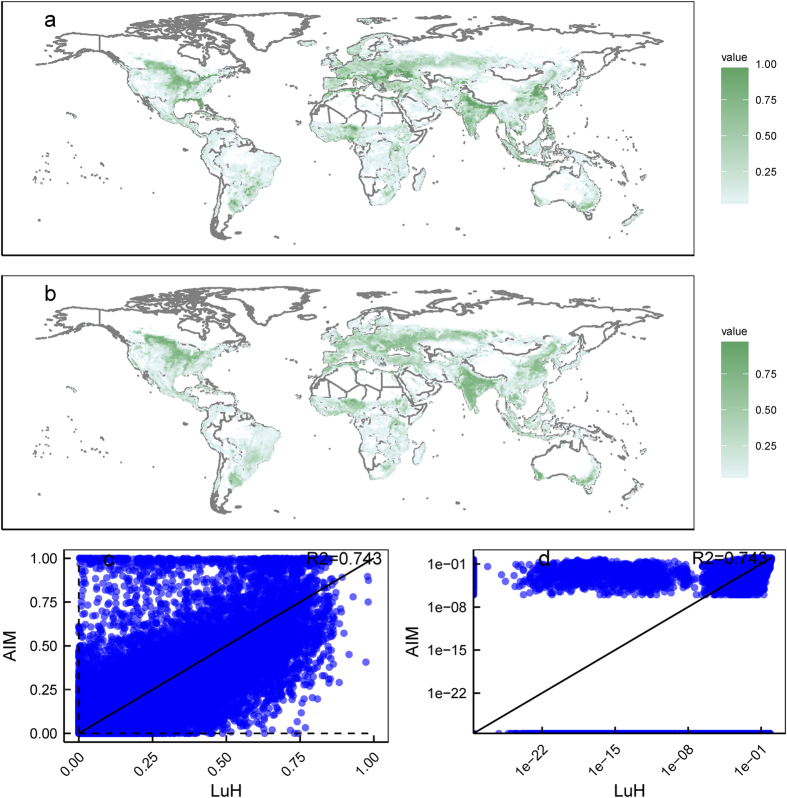
Comparison of downscaled land use in 2005. (**a**) The land-use density for AIM-SSP/RCP. (**b**) The land-use density for LUH2. (**c**) The datasets on normal scales. (**d**) The datasets on logarithmic scales.

Figure 3 presents the cropland area fraction for each grid in AIM-SSP/RCP (Fig. 3a), LUH2 (Fig. 3b), and their comparison (Fig. 3c and d). They showed similar R^2^ values as the emissions results in Fig. 2. We observed several unique characteristics. First, AIM-SSP/RCP had a cut-off below 10^6^, whereas LUH2 had even smaller pixels. Second, some grid cells had AIM-SSP/RCP values around 1 but lower LUH2 values. Third, to obtain data consistent with the regional aggregated AIM/CGE, we reconciled the data using an optimisation model requiring a lower boundary to obtain a numerical solution, as described by Hasegawa *et al.*^13^. The first point was also related to this reconciliation process, mainly to maintain consistency with the regionally aggregated AIM/CGE model output. Another possible reason for the discrepancy between the two datasets is the original source data, since AIM-SSP/RCP uses Monfreda *et al.*^33^ for cropland data, while LUH2 uses HYDE^39^.

## Usage Notes

The data is available at the data repository (Data Citation 1) or (Data Citation 2). As mentioned in the first section, our AIM-SSP/RCP dataset has two main advantages over the ScenarioMIP data: the full coverage of SSPs and RCPs, and the generation of all data by a single IAM. Thus, we believe that this AIM-SSP/RCP could have a complementary role to ScenarioMIP data. These data have multiple applications, several examples of which are outlined below.

### Biodiversity assessment based on isolated socioeconomic assumptions and climate condition factors

Anthropogenic land-use change has had a critical role in historic biodiversity loss, which is expected to continue in the future. Meanwhile, climate is another essential dimension that influences ecosystems, particularly in the future with a wide range of possible climate outcomes. To identify the importance of each factor, biodiversity models may be required to isolate land-use change and climate change factors, which could be performed using the AIM-SSP/RCP dataset. For example, extracting the data for the five SSPs under the same climate conditions would clarify purely socioeconomic effects. ScenarioMIP data do not allow for this application in the sense that it has limited SSP land-use data for each climate mitigation level. More importantly, the land-use data are generated by five IAMs with substantially different mechanisms, making it difficult to interpret differences among SSPs. 7.

### Implications of land use on climate

The most stringent mitigation scenarios include large-scale afforestation and bioenergy crop expansion, which could change the albedo and land surface conditions in terms of climate^40^. For example, heat waves may differ across SSPs even for the same climate mitigation target. Moreover, the climate models may be able to use different land-use sets characterised by SSPs under the same climate target.

Here, we only present examples of biodiversity and local climate implications, and other climate change impact sectors or even non-climate communities may be able to use the data similarly.

Meanwhile, there are several disadvantages and limitations to our AIM-SSP/RCP data.

1. The ScenarioMIP data underwent a data harmonisation process creating almost identical data for the base year across IAMs. Our AIM-SSP/RCP data have not undergone this process, meaning that all scenarios are incompatible with ScenarioMIP data. In addition, it may have some disconnections with CMIP6 historical land-use and emissions data, although the AIM provides the SSP3-Baseline scenario (i.e. SSP3-7.0), which is harmonised with other IAMs as part of the ScenarioMIP data.
2. The classifications and resolutions of the AIM-SSP/RCP data differ from the ScenarioMIP data. For example, ScenarioMIP data have 0.25° spatial resolution, while the resolution of the AIM-SSP/RCP is 0.5°.
3. Since the AIM-SSP/RCP data are generated with a single IAM, we cannot assess the uncertainty associated with IAM representation; however, it is well known that IAM outcomes differ substantially. In the SSP quantification process, this is also described from various perspectives, such as energy^41^, land use^42^, and emissions^43^.

In summary, users of the AIM-SSP/RCP data should understand these advantages and disadvantages.

## Additional information

**How to cite this article:** Fujimori, S. *et al.*, Gridded emissions and land-use data 2005-2100, broad range of socioeconomic and climate mitigation assumptions. *Sci. Data*. 5:180210 doi: 10.1038/sdata.2018.210 (2018).

**Publisher’s note:** Springer Nature remains neutral with regard to jurisdictional claims in published maps and institutional affiliations.

## Supplementary Material



Supplementary File 1

## Figures and Tables

**Table 1:** Regional classifications in the AIM/CGE.

**Code**	**Region**	**Code**	**Region**
JPN	Japan	TUR	Turkey
CHN	China	CAN	Canada
IND	India	USA	United States
XSE	Southeast Asia	BRA	Brazil
XSA	Rest of Asia	XLM	Rest of Latin America
XOC	Oceania	XME	Middle East
XE25	EU25	XNF	North Africa
XER	Rest of Europe	XAF	Rest of Africa
CIS	Former Soviet Union		

**Table 2:** SSP/RCP scenario matrix and scenarios covered in this study.

		**Socioeconomic assumptions**				
		**SSP1**	**SSP2**	**SSP3**	**SSP4**	**SSP5**
Radiative Forcing (Wm−2)	8.5					Baseline
	7.0		Baseline	Baseline		
	6.0	Baseline	X	X	Baseline	X
	4.5	X	X	X	X	X
	3.4	X	X	X	X	X
	2.6	X	X		X	X
	1.9	X	X			
The cells which have X represent the scenarios covered by our dataset. The rest of boxes are either incompatible or were not generated in this study. SSP5-7.0 W is a possible combination, but this forcing level would be too high to be a realistic mitigation target in the context of current policy decisions.						

**Table 3:** Land-use (LU) categories in the land-use allocation model.

**LU class ID**	**LU class name**	**LU class definition**
1	cropland_other	Cropland area, excluding second-generation bioenergy plantations (but including first-generation bioenergy crops); both N-fixing and non-N-fixing; both perennial (e.g. oil palm) and annual
2	cropland_bioenergy	Cropland dedicated to second-generation bioenergy short rotation plantations; perennial cropland
3	grassland	Grassland used for livestock, rangeland or pasture and temporary or permanent
4	forest_unmanaged	Forest areas are not managed, can be both primary and secondary, were present in 2005, but excluding new forest (i.e. afforestation)
5	forest_managed	New areas of managed forest for carbon sequestration (i.e. afforestation).
6	other	Other vegetated (primary or secondary non-forest and non-agricultural vegetation, including shrubland, tundra, or wetlands) and unvegetated (bare land, deserts, inland water, ice, or permanent snow) areas
7	built_up	Built-up areas

**Table 4:** Downscaling algorithm emission source groups and weights.

**Sector**	**Group**	**Weight**
Energy	1	GDP
Industry	1	GDP
Inland transport	1	GDP
Building	1	Population
Solvent	1	GDP
Waste	1	Population
Agriculture	2	
Agricultural waste	2	
Land-use change	2	
Savanna burning	2	
International navigation	3	
Aviation	3	

**Table 5:** Naming convention of the emissions and land-use data file.

	**Elements of each dimension**	**File name**
**Socioeconomic assumption**	SSP1	SSP1
	SSP2	SSP2
	SSP3	SSP3
	SSP4	SSP4
	SSP5	SSP5
**Climate mitigation level**	Baseline	Baseline
	6.0 Wm^−2^	60
	4.5 Wm^−2^	45
	3.4 Wm^−2^	34
	2.6 Wm^−2^	26
	1.9 Wm^−2^	19
**Species**	BC	BCE
	CH_4_	CH4
	CO	COE
	NH_3_	NH3
	NO_X_	NOX
	OC	OCE
	Sulphur	SO2
	VOC	VOC

**Table 6:** Emissions sector naming convention.

**Sector**	**Variable name**
Energy	emiss_ene
Industry	emiss_ind
Inland transport	emiss_tra
Building	emiss_dom
Solvent	emiss_slv
Waste	emiss_wst
Agriculture	emiss_agr
Agricultural waste	emiss_awb
Land-use change	emiss_lcf
Savanna burning	emiss_sav
International navigation	emiss_shp
Aviation	emiss_air

**Table 7:** Regionally aggregated and global total emissions for AIM-SSP/RCP(AIM) and CEDS across different sectors.

		**Building**			**Energy**			**Industry**			**Transport**		
		**AIM**	**CEDS**	**Ratio**	**AIM**	**CEDS**	**Ratio**	**AIM**	**CEDS**	**Ratio**	**AIM**	**CEDS**	**Ratio**
SO_2_	BRA	0.09	0.08	1.10	0.34	0.23	1.48	0.89	1.12	0.79	0.16	0.25	0.63
	CAN	0.30	0.06	5.39	0.86	1.08	0.80	0.97	1.05	0.92	0.11	0.09	1.25
	CHN	2.03	2.89	0.70	18.17	15.43	1.18	9.82	11.43	0.86	0.69	0.47	1.46
	CIS	0.78	0.51	1.51	4.34	5.47	0.79	4.95	4.74	1.04	0.14	0.17	0.85
	IND	0.67	0.70	0.96	4.36	4.63	0.94	1.68	2.30	0.73	0.15	0.20	0.74
	JPN	0.87	0.03	32.62	0.67	0.16	4.14	0.95	0.40	2.37	0.24	0.18	1.29
	TUR	0.15	0.16	0.89	0.62	1.28	0.49	0.52	0.57	0.91	0.05	0.07	0.66
	USA	0.16	0.80	0.20	8.92	10.17	0.88	1.66	2.00	0.83	0.20	0.46	0.45
	XAF	0.50	0.27	1.85	2.03	1.94	1.04	1.04	1.19	0.88	0.10	0.20	0.51
	XE25	1.02	0.71	1.43	5.28	4.85	1.09	2.04	1.51	1.34	0.37	0.18	2.04
	XER	0.12	0.07	1.65	1.03	2.30	0.45	0.32	0.29	1.13	0.05	0.06	0.88
	XLM	0.37	0.12	3.18	3.27	2.71	1.20	2.99	2.61	1.14	0.34	0.43	0.80
	XME	0.31	0.17	1.77	4.19	4.69	0.89	1.41	1.71	0.82	0.20	0.65	0.31
	XNF	0.14	0.03	4.57	0.85	0.40	2.13	0.38	0.44	0.87	0.10	0.24	0.40
	XOC	0.07	0.01	5.18	0.69	0.64	1.08	2.00	0.80	2.50	0.04	0.04	0.94
	XSA	0.13	0.12	1.05	0.61	0.50	1.23	0.24	0.39	0.60	0.03	0.21	0.16
	XSE	0.73	0.40	1.84	3.91	2.40	1.63	3.08	1.58	1.95	0.48	0.40	1.18
	Total	8.43	7.14	1.18	60.14	58.87	1.02	34.93	34.14	1.02	3.46	4.31	0.80
NO_X_	BRA	0.06	0.06	1.00	0.30	0.25	1.21	0.42	0.43	0.97	1.19	1.18	1.00
	CAN	0.12	0.37	0.31	0.43	0.83	0.52	0.27	0.32	0.86	0.78	1.04	0.75
	CHN	0.89	1.23	0.73	8.31	8.21	1.01	3.70	4.39	0.84	3.33	5.79	0.57
	CIS	0.42	0.38	1.10	3.32	3.40	0.98	0.57	0.71	0.81	1.66	1.80	0.93
	IND	0.44	0.84	0.52	2.86	2.49	1.15	0.79	0.59	1.34	1.38	3.06	0.45
	JPN	0.20	0.29	0.69	0.79	0.62	1.28	0.57	0.43	1.31	0.92	1.98	0.46
	TUR	0.06	0.18	0.36	0.22	0.24	0.90	0.15	0.11	1.37	0.40	0.33	1.23
	USA	0.76	1.90	0.40	5.63	4.66	1.21	1.38	2.24	0.62	7.36	10.02	0.73
	XAF	0.43	0.54	0.80	1.08	1.00	1.08	0.28	0.40	0.69	1.02	0.98	1.04
	XE25	0.95	0.73	1.31	3.90	2.41	1.62	1.26	1.37	0.92	4.44	5.88	0.76
	XER	0.07	0.06	1.18	0.31	0.39	0.79	0.13	0.21	0.61	0.49	0.47	1.04
	XLM	0.18	0.39	0.45	1.41	1.32	1.07	0.46	0.57	0.81	2.75	2.66	1.03
	XME	0.18	0.16	1.10	1.93	1.74	1.11	0.42	0.48	0.88	2.63	2.39	1.10
	XNF	0.08	0.05	1.57	0.42	0.37	1.14	0.13	0.14	0.92	0.53	0.56	0.95
	XOC	0.03	0.03	0.79	0.69	0.60	1.14	0.15	0.20	0.73	0.39	0.44	0.90
	XSA	0.11	0.33	0.33	0.31	0.25	1.26	0.14	0.12	1.10	0.47	0.61	0.78
	XSE	0.38	0.56	0.68	1.90	1.55	1.22	1.07	0.91	1.18	2.99	2.81	1.06
	Total	5.35	8.11	0.66	33.81	30.34	1.11	11.89	13.63	0.87	32.72	41.99	0.78
The third column for each sector shows the ratio of AIM-SSP/RCP to CEDS values.													

**Table 8:** Regression estimates (αr: regional parameters; c: intercept).

		**Building**	**Energy**	**Industry**	**Transport**
SO_2_	(Intercept)	0.49	3.28	0.07	0.01
	CAN	−0.67	−3.29	0.27	−1.49
	CHN	−0.34	−2.60	−0.43	0.30
	CIS	−0.23	−1.97	0.41	-0.56
	IND	−0.41	−1.51	−0.16	−0.13
	JPN	0.32	−0.73	−0.76	−0.23
	TUR	−0.10	−3.12	−0.07	−0.21
	USA	−0.81	−1.82	−0.23	−1.11
	XAF	−0.47	−1.83	0.00	−0.12
	XE25	−0.18	−2.09	−0.43	0.02
	XER	−0.27	−1.46	−0.33	−0.20
	XLM	0.07	−2.36	−0.03	0.01
	XME	0.64	1.57	−0.07	−0.27
	XNF	0.11	−2.24	−0.07	−0.27
	XOC	0.75	−1.83	−0.11	0.00
	XSA	−0.35	−1.15	−0.07	−0.43
	XSE	−0.64	−1.28	−0.12	0.35
NOx	(Intercept)	0.30	0.01	0.57	0.09
	CAN	−0.48	0.42	−0.12	−1.70
	CHN	−0.36	0.55	−0.75	0.22
	CIS	0.04	1.49	0.14	−0.25
	IND	−0.75	1.82	−0.55	−0.31
	JPN	-0.79	0.54	-1.02	-0.61
	TUR	−0.39	0.03	−0.57	0.03
	USA	−0.33	1.67	−0.55	−0.89
	XAF	−1.05	1.39	−0.48	0.07
	XE25	−0.07	1.65	−0.75	−0.32
	XER	0.02	0.51	−0.68	−0.43
	XLM	−0.42	0.43	−0.35	0.01
	XME	0.24	1.67	−0.57	0.14
	XNF	−0.14	1.62	−0.57	−0.02
	XOC	0.31	1.46	−0.56	0.45
	XSA	−0.38	1.07	−0.49	−0.02
	XSE	−0.43	1.15	−0.22	0.02
The regional codes are shown in Table 1. Columns are sectors.					
