# Supplementary material for: Gridded emissions and land-use data for 2005–2100 under diverse socioeconomic and climate mitigation scenarios
Source: Sci Data. 2018 Oct 16;5:180210. doi: 10.1038/sdata.2018.210 (PMC6190744; doi:10.1038/sdata.2018.210)
Supplement: Supplementary File 1 [file sdata2018210-s2.docx]

**Supplementary File 1**

**Table of contents**

Supplementary Tables

[Supplementary Table S 1: Industrial classification 2](#_Toc521501312)

[Supplementary Table S 2: Full list of emissions files 3](#_Toc521501313)

[Supplementary Table S 3: Full list of land-use files 8](#_Toc521501314)

Supplementary Figures

[Supplementary Figure S 1: Geographic regions in the AIM/CGE model. See Table 2 for the definitions of the regional codes. 9](#_Toc521501300)

[Supplementary Figure S 2: Comparison of downscaled NO_X_ emissions from the energy sector in 2010. 10](#_Toc521501301)

[Supplementary Figure S 3: Comparison of the downscaled NO_X_ emissions energy sector in 2010. 11](#_Toc521501302)

[Supplementary Figure S 4: Comparison of the downscaled SO_2_ emissions energy sector in 2010. 12](#_Toc521501303)

[Supplementary Figure S 5: Comparison of the downscaled SO_2_ emissions from the transport sector in 2005. 13](#_Toc521501304)

[Supplementary Figure S 6: Comparison of the downscaled SO_2_ emissions from the residential and commercial sector in 2005. 14](#_Toc521501305)

[Supplementary Figure S 7: Comparison of the downscaled SO_2_ emissions from the energy sector in 2015. 15](#_Toc521501306)

[Supplementary Figure S 8: Comparison of the downscaled SO_2_ emissions from the energy sector in 2050. 16](#_Toc521501307)

[Supplementary Figure S 9: Comparison of the downscaled SO_2_ emissions from the energy sector in 2100. 17](#_Toc521501308)

[Supplementary Figure S 10: Comparison of the downscaled NOx emissions from the energy sector in 2015. 18](#_Toc521501309)

[Supplementary Figure S 11: Comparison of the downscaled NOx emissions from the energy sector in 2050. 19](#_Toc521501310)

[Supplementary Figure S 12: Comparison of the downscaled NOx emissions from the energy sector in 2100. 20](#_Toc521501311)

Codes for the data generation

Emissions: “AIMGridemissioncode.egg”

Land-use: “AIMGridLandusecode.egg”

Supplementary Table S 1: Industrial classification

| Agricultural sectors | Energy supply sectors | Other production sectors |
| --- | --- | --- |
| Rice | Coal mining | Mineral mining and other quarrying |
| Wheat | Oil mining | Food products |
| Other grains | Gas mining | Textiles, apparel, and leather |
| Oil seed crops | Petroleum refinery | Wood products |
| Sugar crops | Coal transformation | Paper, paper products, and pulp |
| Other crops | Biomass transformation (first generation) | Chemical, plastic, and rubber products |
| Ruminant livestock | Biomass transformation (second generation with energy crops) | Iron and steel |
| Raw milk | Biomass transformation (second generation with residues) | Nonferrous products |
| Other livestock and fisheries | Gas distribution | Other manufacturing |
| Forestry | Coal-fired power | Construction |
|  | Oil-fired power | Transport and communications |
|  | Gas-fired power | Other service sectors |
|  | Nuclear power | CCS [Please define] service |
|  | Hydroelectric power |  |
|  | Geothermal power |  |
|  | Photovoltaic power |  |
|  | Wind power |  |
|  | Waste biomass power |  |
|  | Other renewable energy power generation |  |
|  | Advanced biomass-power generation |  |

Supplementary Table S 2: Full list of emissions files

| File name | Socioeconomic assumption | Climate mitigation level | Species |
| --- | --- | --- | --- |
| AIM-SSPRCP-Emission-SSP1-19-BCE-v.1.0.nc | SSP1 | 1.9 Wm^−2^ | BC |
| AIM-SSPRCP-Emission-SSP1-19-CH4-v.1.0.nc | SSP1 | 1.9 Wm^−2^ | CH_4_ |
| AIM-SSPRCP-Emission-SSP1-19-COE-v.1.0.nc | SSP1 | 1.9 Wm^−2^ | CO |
| AIM-SSPRCP-Emission-SSP1-19-NH3-v.1.0.nc | SSP1 | 1.9 Wm^−2^ | NH_3_ |
| AIM-SSPRCP-Emission-SSP1-19-NOX-v.1.0.nc | SSP1 | 1.9 Wm^−2^ | NO_X_ |
| AIM-SSPRCP-Emission-SSP1-19-OCE-v.1.0.nc | SSP1 | 1.9 Wm^−2^ | OC |
| AIM-SSPRCP-Emission-SSP1-19-SO2-v.1.0.nc | SSP1 | 1.9 Wm^−2^ | Sulphur |
| AIM-SSPRCP-Emission-SSP1-19-VOC-v.1.0.nc | SSP1 | 1.9 Wm^−2^ | VOC |
| AIM-SSPRCP-Emission-SSP1-26-BCE-v.1.0.nc | SSP1 | 2.6 Wm^−2^ | BC |
| AIM-SSPRCP-Emission-SSP1-26-CH4-v.1.0.nc | SSP1 | 2.6 Wm^−2^ | CH_4_ |
| AIM-SSPRCP-Emission-SSP1-26-COE-v.1.0.nc | SSP1 | 2.6 Wm^−2^ | CO |
| AIM-SSPRCP-Emission-SSP1-26-NH3-v.1.0.nc | SSP1 | 2.6 Wm^−2^ | NH_3_ |
| AIM-SSPRCP-Emission-SSP1-26-NOX-v.1.0.nc | SSP1 | 2.6 Wm^−2^ | NO_X_ |
| AIM-SSPRCP-Emission-SSP1-26-OCE-v.1.0.nc | SSP1 | 2.6 Wm^−2^ | OC |
| AIM-SSPRCP-Emission-SSP1-26-SO2-v.1.0.nc | SSP1 | 2.6 Wm^−2^ | Sulphur |
| AIM-SSPRCP-Emission-SSP1-26-VOC-v.1.0.nc | SSP1 | 2.6 Wm^−2^ | VOC |
| AIM-SSPRCP-Emission-SSP1-34-BCE-v.1.0.nc | SSP1 | 3.4 Wm^−2^ | BC |
| AIM-SSPRCP-Emission-SSP1-34-CH4-v.1.0.nc | SSP1 | 3.4 Wm^−2^ | CH_4_ |
| AIM-SSPRCP-Emission-SSP1-34-COE-v.1.0.nc | SSP1 | 3.4 Wm^−2^ | CO |
| AIM-SSPRCP-Emission-SSP1-34-NH3-v.1.0.nc | SSP1 | 3.4 Wm^−2^ | NH_3_ |
| AIM-SSPRCP-Emission-SSP1-34-NOX-v.1.0.nc | SSP1 | 3.4 Wm^−2^ | NO_X_ |
| AIM-SSPRCP-Emission-SSP1-34-OCE-v.1.0.nc | SSP1 | 3.4 Wm^−2^ | OC |
| AIM-SSPRCP-Emission-SSP1-34-SO2-v.1.0.nc | SSP1 | 3.4 Wm^−2^ | Sulphur |
| AIM-SSPRCP-Emission-SSP1-34-VOC-v.1.0.nc | SSP1 | 3.4 Wm^−2^ | VOC |
| AIM-SSPRCP-Emission-SSP1-45-BCE-v.1.0.nc | SSP1 | 4.5 Wm^−2^ | BC |
| AIM-SSPRCP-Emission-SSP1-45-CH4-v.1.0.nc | SSP1 | 4.5 Wm^−2^ | CH_4_ |
| AIM-SSPRCP-Emission-SSP1-45-COE-v.1.0.nc | SSP1 | 4.5 Wm^−2^ | CO |
| AIM-SSPRCP-Emission-SSP1-45-NH3-v.1.0.nc | SSP1 | 4.5 Wm^−2^ | NH_3_ |
| AIM-SSPRCP-Emission-SSP1-45-NOX-v.1.0.nc | SSP1 | 4.5 Wm^−2^ | NO_X_ |
| AIM-SSPRCP-Emission-SSP1-45-OCE-v.1.0.nc | SSP1 | 4.5 Wm^−2^ | OC |
| AIM-SSPRCP-Emission-SSP1-45-SO2-v.1.0.nc | SSP1 | 4.5 Wm^−2^ | Sulphur |
| AIM-SSPRCP-Emission-SSP1-45-VOC-v.1.0.nc | SSP1 | 4.5 Wm^−2^ | VOC |
| AIM-SSPRCP-Emission-SSP1-Baseline-BCE-v.1.0.nc | SSP1 | Baseline | BC |
| AIM-SSPRCP-Emission-SSP1-Baseline-CH4-v.1.0.nc | SSP1 | Baseline | CH_4_ |
| AIM-SSPRCP-Emission-SSP1-Baseline-COE-v.1.0.nc | SSP1 | Baseline | CO |
| AIM-SSPRCP-Emission-SSP1-Baseline-NH3-v.1.0.nc | SSP1 | Baseline | NH_3_ |
| AIM-SSPRCP-Emission-SSP1-Baseline-NOX-v.1.0.nc | SSP1 | Baseline | NO_X_ |
| AIM-SSPRCP-Emission-SSP1-Baseline-OCE-v.1.0.nc | SSP1 | Baseline | OC |
| AIM-SSPRCP-Emission-SSP1-Baseline-SO2-v.1.0.nc | SSP1 | Baseline | Sulphur |
| AIM-SSPRCP-Emission-SSP1-Baseline-VOC-v.1.0.nc | SSP1 | Baseline | VOC |
| AIM-SSPRCP-Emission-SSP2-19-BCE-v.1.0.nc | SSP2 | 1.9 Wm^−2^ | BC |
| AIM-SSPRCP-Emission-SSP2-19-CH4-v.1.0.nc | SSP2 | 1.9 Wm^−2^ | CH_4_ |
| AIM-SSPRCP-Emission-SSP2-19-COE-v.1.0.nc | SSP2 | 1.9 Wm^−2^ | CO |
| AIM-SSPRCP-Emission-SSP2-19-NH3-v.1.0.nc | SSP2 | 1.9 Wm^−2^ | NH_3_ |
| AIM-SSPRCP-Emission-SSP2-19-NOX-v.1.0.nc | SSP2 | 1.9 Wm^−2^ | NO_X_ |
| AIM-SSPRCP-Emission-SSP2-19-OCE-v.1.0.nc | SSP2 | 1.9 Wm^−2^ | OC |
| AIM-SSPRCP-Emission-SSP2-19-SO2-v.1.0.nc | SSP2 | 1.9 Wm^−2^ | Sulphur |
| AIM-SSPRCP-Emission-SSP2-19-VOC-v.1.0.nc | SSP2 | 1.9 Wm^−2^ | VOC |
| AIM-SSPRCP-Emission-SSP2-26-BCE-v.1.0.nc | SSP2 | 2.6 Wm^−2^ | BC |
| AIM-SSPRCP-Emission-SSP2-26-CH4-v.1.0.nc | SSP2 | 2.6 Wm^−2^ | CH_4_ |
| AIM-SSPRCP-Emission-SSP2-26-COE-v.1.0.nc | SSP2 | 2.6 Wm^−2^ | CO |
| AIM-SSPRCP-Emission-SSP2-26-NH3-v.1.0.nc | SSP2 | 2.6 Wm^−2^ | NH_3_ |
| AIM-SSPRCP-Emission-SSP2-26-NOX-v.1.0.nc | SSP2 | 2.6 Wm^−2^ | NO_X_ |
| AIM-SSPRCP-Emission-SSP2-26-OCE-v.1.0.nc | SSP2 | 2.6 Wm^−2^ | OC |
| AIM-SSPRCP-Emission-SSP2-26-SO2-v.1.0.nc | SSP2 | 2.6 Wm^−2^ | Sulphur |
| AIM-SSPRCP-Emission-SSP2-26-VOC-v.1.0.nc | SSP2 | 2.6 Wm^−2^ | VOC |
| AIM-SSPRCP-Emission-SSP2-34-BCE-v.1.0.nc | SSP2 | 3.4 Wm^−2^ | BC |
| AIM-SSPRCP-Emission-SSP2-34-CH4-v.1.0.nc | SSP2 | 3.4 Wm^−2^ | CH_4_ |
| AIM-SSPRCP-Emission-SSP2-34-COE-v.1.0.nc | SSP2 | 3.4 Wm^−2^ | CO |
| AIM-SSPRCP-Emission-SSP2-34-NH3-v.1.0.nc | SSP2 | 3.4 Wm^−2^ | NH_3_ |
| AIM-SSPRCP-Emission-SSP2-34-NOX-v.1.0.nc | SSP2 | 3.4 Wm^−2^ | NO_X_ |
| AIM-SSPRCP-Emission-SSP2-34-OCE-v.1.0.nc | SSP2 | 3.4 Wm^−2^ | OC |
| AIM-SSPRCP-Emission-SSP2-34-SO2-v.1.0.nc | SSP2 | 3.4 Wm^−2^ | Sulphur |
| AIM-SSPRCP-Emission-SSP2-34-VOC-v.1.0.nc | SSP2 | 3.4 Wm^−2^ | VOC |
| AIM-SSPRCP-Emission-SSP2-45-BCE-v.1.0.nc | SSP2 | 4.5 Wm^−2^ | BC |
| AIM-SSPRCP-Emission-SSP2-45-CH4-v.1.0.nc | SSP2 | 4.5 Wm^−2^ | CH_4_ |
| AIM-SSPRCP-Emission-SSP2-45-COE-v.1.0.nc | SSP2 | 4.5 Wm^−2^ | CO |
| AIM-SSPRCP-Emission-SSP2-45-NH3-v.1.0.nc | SSP2 | 4.5 Wm^−2^ | NH_3_ |
| AIM-SSPRCP-Emission-SSP2-45-NOX-v.1.0.nc | SSP2 | 4.5 Wm^−2^ | NO_X_ |
| AIM-SSPRCP-Emission-SSP2-45-OCE-v.1.0.nc | SSP2 | 4.5 Wm^−2^ | OC |
| AIM-SSPRCP-Emission-SSP2-45-SO2-v.1.0.nc | SSP2 | 4.5 Wm^−2^ | Sulphur |
| AIM-SSPRCP-Emission-SSP2-45-VOC-v.1.0.nc | SSP2 | 4.5 Wm^−2^ | VOC |
| AIM-SSPRCP-Emission-SSP2-60-BCE-v.1.0.nc | SSP2 | 6.0 Wm^−2^ | BC |
| AIM-SSPRCP-Emission-SSP2-60-CH4-v.1.0.nc | SSP2 | 6.0 Wm^−2^ | CH_4_ |
| AIM-SSPRCP-Emission-SSP2-60-COE-v.1.0.nc | SSP2 | 6.0 Wm^−2^ | CO |
| AIM-SSPRCP-Emission-SSP2-60-NH3-v.1.0.nc | SSP2 | 6.0 Wm^−2^ | NH_3_ |
| AIM-SSPRCP-Emission-SSP2-60-NOX-v.1.0.nc | SSP2 | 6.0 Wm^−2^ | NO_X_ |
| AIM-SSPRCP-Emission-SSP2-60-OCE-v.1.0.nc | SSP2 | 6.0 Wm^−2^ | OC |
| AIM-SSPRCP-Emission-SSP2-60-SO2-v.1.0.nc | SSP2 | 6.0 Wm^−2^ | Sulphur |
| AIM-SSPRCP-Emission-SSP2-60-VOC-v.1.0.nc | SSP2 | 6.0 Wm^−2^ | VOC |
| AIM-SSPRCP-Emission-SSP2-Baseline-BCE-v.1.0.nc | SSP2 | Baseline | BC |
| AIM-SSPRCP-Emission-SSP2-Baseline-CH4-v.1.0.nc | SSP2 | Baseline | CH_4_ |
| AIM-SSPRCP-Emission-SSP2-Baseline-COE-v.1.0.nc | SSP2 | Baseline | CO |
| AIM-SSPRCP-Emission-SSP2-Baseline-NH3-v.1.0.nc | SSP2 | Baseline | NH_3_ |
| AIM-SSPRCP-Emission-SSP2-Baseline-NOX-v.1.0.nc | SSP2 | Baseline | NO_X_ |
| AIM-SSPRCP-Emission-SSP2-Baseline-OCE-v.1.0.nc | SSP2 | Baseline | OC |
| AIM-SSPRCP-Emission-SSP2-Baseline-SO2-v.1.0.nc | SSP2 | Baseline | Sulphur |
| AIM-SSPRCP-Emission-SSP2-Baseline-VOC-v.1.0.nc | SSP2 | Baseline | VOC |
| AIM-SSPRCP-Emission-SSP3-34-BCE-v.1.0.nc | SSP1 | 3.4 Wm^−2^ | BC |
| AIM-SSPRCP-Emission-SSP3-34-CH4-v.1.0.nc | SSP1 | 3.4 Wm^−2^ | CH_4_ |
| AIM-SSPRCP-Emission-SSP3-34-COE-v.1.0.nc | SSP1 | 3.4 Wm^−2^ | CO |
| AIM-SSPRCP-Emission-SSP3-34-NH3-v.1.0.nc | SSP1 | 3.4 Wm^−2^ | NH_3_ |
| AIM-SSPRCP-Emission-SSP3-34-NOX-v.1.0.nc | SSP1 | 3.4 Wm^−2^ | NO_X_ |
| AIM-SSPRCP-Emission-SSP3-34-OCE-v.1.0.nc | SSP1 | 3.4 Wm^−2^ | OC |
| AIM-SSPRCP-Emission-SSP3-34-SO2-v.1.0.nc | SSP1 | 3.4 Wm^−2^ | Sulphur |
| AIM-SSPRCP-Emission-SSP3-34-VOC-v.1.0.nc | SSP1 | 3.4 Wm^−2^ | VOC |
| AIM-SSPRCP-Emission-SSP3-45-BCE-v.1.0.nc | SSP1 | 4.5 Wm^−2^ | BC |
| AIM-SSPRCP-Emission-SSP3-45-CH4-v.1.0.nc | SSP1 | 4.5 Wm^−2^ | CH_4_ |
| AIM-SSPRCP-Emission-SSP3-45-COE-v.1.0.nc | SSP1 | 4.5 Wm^−2^ | CO |
| AIM-SSPRCP-Emission-SSP3-45-NH3-v.1.0.nc | SSP1 | 4.5 Wm^−2^ | NH_3_ |
| AIM-SSPRCP-Emission-SSP3-45-NOX-v.1.0.nc | SSP1 | 4.5 Wm^−2^ | NO_X_ |
| AIM-SSPRCP-Emission-SSP3-45-OCE-v.1.0.nc | SSP1 | 4.5 Wm^−2^ | OC |
| AIM-SSPRCP-Emission-SSP3-45-SO2-v.1.0.nc | SSP1 | 4.5 Wm^−2^ | Sulphur |
| AIM-SSPRCP-Emission-SSP3-45-VOC-v.1.0.nc | SSP1 | 4.5 Wm^−2^ | VOC |
| AIM-SSPRCP-Emission-SSP3-60-BCE-v.1.0.nc | SSP3 | 6.0 Wm^−2^ | BC |
| AIM-SSPRCP-Emission-SSP3-60-CH4-v.1.0.nc | SSP3 | 6.0 Wm^−2^ | CH_4_ |
| AIM-SSPRCP-Emission-SSP3-60-COE-v.1.0.nc | SSP3 | 6.0 Wm^−2^ | CO |
| AIM-SSPRCP-Emission-SSP3-60-NH3-v.1.0.nc | SSP3 | 6.0 Wm^−2^ | NH_3_ |
| AIM-SSPRCP-Emission-SSP3-60-NOX-v.1.0.nc | SSP3 | 6.0 Wm^−2^ | NO_X_ |
| AIM-SSPRCP-Emission-SSP3-60-OCE-v.1.0.nc | SSP3 | 6.0 Wm^−2^ | OC |
| AIM-SSPRCP-Emission-SSP3-60-SO2-v.1.0.nc | SSP3 | 6.0 Wm^−2^ | Sulphur |
| AIM-SSPRCP-Emission-SSP3-60-VOC-v.1.0.nc | SSP3 | 6.0 Wm^−2^ | VOC |
| AIM-SSPRCP-Emission-SSP3-Baseline-BCE-v.1.0.nc | SSP3 | Baseline | BC |
| AIM-SSPRCP-Emission-SSP3-Baseline-CH4-v.1.0.nc | SSP3 | Baseline | CH_4_ |
| AIM-SSPRCP-Emission-SSP3-Baseline-COE-v.1.0.nc | SSP3 | Baseline | CO |
| AIM-SSPRCP-Emission-SSP3-Baseline-NH3-v.1.0.nc | SSP3 | Baseline | NH_3_ |
| AIM-SSPRCP-Emission-SSP3-Baseline-NOX-v.1.0.nc | SSP3 | Baseline | NO_X_ |
| AIM-SSPRCP-Emission-SSP3-Baseline-OCE-v.1.0.nc | SSP3 | Baseline | OC |
| AIM-SSPRCP-Emission-SSP3-Baseline-SO2-v.1.0.nc | SSP3 | Baseline | Sulphur |
| AIM-SSPRCP-Emission-SSP3-Baseline-VOC-v.1.0.nc | SSP3 | Baseline | VOC |
| AIM-SSPRCP-Emission-SSP4-26-BCE-v.1.0.nc | SSP4 | 2.6 Wm^−2^ | BC |
| AIM-SSPRCP-Emission-SSP4-26-CH4-v.1.0.nc | SSP4 | 2.6 Wm^−2^ | CH_4_ |
| AIM-SSPRCP-Emission-SSP4-26-COE-v.1.0.nc | SSP4 | 2.6 Wm^−2^ | CO |
| AIM-SSPRCP-Emission-SSP4-26-NH3-v.1.0.nc | SSP4 | 2.6 Wm^−2^ | NH_3_ |
| AIM-SSPRCP-Emission-SSP4-26-NOX-v.1.0.nc | SSP4 | 2.6 Wm^−2^ | NO_X_ |
| AIM-SSPRCP-Emission-SSP4-26-OCE-v.1.0.nc | SSP4 | 2.6 Wm^−2^ | OC |
| AIM-SSPRCP-Emission-SSP4-26-SO2-v.1.0.nc | SSP4 | 2.6 Wm^−2^ | Sulphur |
| AIM-SSPRCP-Emission-SSP4-26-VOC-v.1.0.nc | SSP4 | 2.6 Wm^−2^ | VOC |
| AIM-SSPRCP-Emission-SSP4-34-BCE-v.1.0.nc | SSP4 | 3.4 Wm^−2^ | BC |
| AIM-SSPRCP-Emission-SSP4-34-CH4-v.1.0.nc | SSP4 | 3.4 Wm^−2^ | CH_4_ |
| AIM-SSPRCP-Emission-SSP4-34-COE-v.1.0.nc | SSP4 | 3.4 Wm^−2^ | CO |
| AIM-SSPRCP-Emission-SSP4-34-NH3-v.1.0.nc | SSP4 | 3.4 Wm^−2^ | NH_3_ |
| AIM-SSPRCP-Emission-SSP4-34-NOX-v.1.0.nc | SSP4 | 3.4 Wm^−2^ | NO_X_ |
| AIM-SSPRCP-Emission-SSP4-34-OCE-v.1.0.nc | SSP4 | 3.4 Wm^−2^ | OC |
| AIM-SSPRCP-Emission-SSP4-34-SO2-v.1.0.nc | SSP4 | 3.4 Wm^−2^ | Sulphur |
| AIM-SSPRCP-Emission-SSP4-34-VOC-v.1.0.nc | SSP4 | 3.4 Wm^−2^ | VOC |
| AIM-SSPRCP-Emission-SSP4-45-BCE-v.1.0.nc | SSP4 | 4.5 Wm^−2^ | BC |
| AIM-SSPRCP-Emission-SSP4-45-CH4-v.1.0.nc | SSP4 | 4.5 Wm^−2^ | CH_4_ |
| AIM-SSPRCP-Emission-SSP4-45-COE-v.1.0.nc | SSP4 | 4.5 Wm^−2^ | CO |
| AIM-SSPRCP-Emission-SSP4-45-NH3-v.1.0.nc | SSP4 | 4.5 Wm^−2^ | NH_3_ |
| AIM-SSPRCP-Emission-SSP4-45-NOX-v.1.0.nc | SSP4 | 4.5 Wm^−2^ | NO_X_ |
| AIM-SSPRCP-Emission-SSP4-45-OCE-v.1.0.nc | SSP4 | 4.5 Wm^−2^ | OC |
| AIM-SSPRCP-Emission-SSP4-45-SO2-v.1.0.nc | SSP4 | 4.5 Wm^−2^ | Sulphur |
| AIM-SSPRCP-Emission-SSP4-45-VOC-v.1.0.nc | SSP4 | 4.5 Wm^−2^ | VOC |
| AIM-SSPRCP-Emission-SSP4-Baseline-BCE-v.1.0.nc | SSP4 | Baseline | BC |
| AIM-SSPRCP-Emission-SSP4-Baseline-CH4-v.1.0.nc | SSP4 | Baseline | CH_4_ |
| AIM-SSPRCP-Emission-SSP4-Baseline-COE-v.1.0.nc | SSP4 | Baseline | CO |
| AIM-SSPRCP-Emission-SSP4-Baseline-NH3-v.1.0.nc | SSP4 | Baseline | NH_3_ |
| AIM-SSPRCP-Emission-SSP4-Baseline-NOX-v.1.0.nc | SSP4 | Baseline | NO_X_ |
| AIM-SSPRCP-Emission-SSP4-Baseline-OCE-v.1.0.nc | SSP4 | Baseline | OC |
| AIM-SSPRCP-Emission-SSP4-Baseline-SO2-v.1.0.nc | SSP4 | Baseline | Sulphur |
| AIM-SSPRCP-Emission-SSP4-Baseline-VOC-v.1.0.nc | SSP4 | Baseline | VOC |
| AIM-SSPRCP-Emission-SSP5-26-BCE-v.1.0.nc | SSP5 | 2.6 Wm^−2^ | BC |
| AIM-SSPRCP-Emission-SSP5-26-CH4-v.1.0.nc | SSP5 | 2.6 Wm^−2^ | CH_4_ |
| AIM-SSPRCP-Emission-SSP5-26-COE-v.1.0.nc | SSP5 | 2.6 Wm^−2^ | CO |
| AIM-SSPRCP-Emission-SSP5-26-NH3-v.1.0.nc | SSP5 | 2.6 Wm^−2^ | NH_3_ |
| AIM-SSPRCP-Emission-SSP5-26-NOX-v.1.0.nc | SSP5 | 2.6 Wm^−2^ | NO_X_ |
| AIM-SSPRCP-Emission-SSP5-26-OCE-v.1.0.nc | SSP5 | 2.6 Wm^-2^ | OC |
| AIM-SSPRCP-Emission-SSP5-26-SO2-v.1.0.nc | SSP5 | 2.6 Wm^−2^ | Sulphur |
| AIM-SSPRCP-Emission-SSP5-26-VOC-v.1.0.nc | SSP5 | 2.6 Wm^−2^ | VOC |
| AIM-SSPRCP-Emission-SSP5-34-BCE-v.1.0.nc | SSP5 | 3.4 Wm^−2^ | BC |
| AIM-SSPRCP-Emission-SSP5-34-CH4-v.1.0.nc | SSP5 | 3.4 Wm^−2^ | CH_4_ |
| AIM-SSPRCP-Emission-SSP5-34-COE-v.1.0.nc | SSP5 | 3.4 Wm^−2^ | CO |
| AIM-SSPRCP-Emission-SSP5-34-NH3-v.1.0.nc | SSP5 | 3.4 Wm^−2^ | NH_3_ |
| AIM-SSPRCP-Emission-SSP5-34-NOX-v.1.0.nc | SSP5 | 3.4 Wm^−2^ | NO_X_ |
| AIM-SSPRCP-Emission-SSP5-34-OCE-v.1.0.nc | SSP5 | 3.4 Wm^−2^ | OC |
| AIM-SSPRCP-Emission-SSP5-34-SO2-v.1.0.nc | SSP5 | 3.4 Wm^−2^ | Sulphur |
| AIM-SSPRCP-Emission-SSP5-34-VOC-v.1.0.nc | SSP5 | 3.4 Wm^−2^ | VOC |
| AIM-SSPRCP-Emission-SSP5-45-BCE-v.1.0.nc | SSP5 | 4.5 Wm^−2^ | BC |
| AIM-SSPRCP-Emission-SSP5-45-CH4-v.1.0.nc | SSP5 | 4.5 Wm^−2^ | CH_4_ |
| AIM-SSPRCP-Emission-SSP5-45-COE-v.1.0.nc | SSP5 | 4.5 Wm^−2^ | CO |
| AIM-SSPRCP-Emission-SSP5-45-NH3-v.1.0.nc | SSP5 | 4.5 Wm^−2^ | NH_3_ |
| AIM-SSPRCP-Emission-SSP5-45-NOX-v.1.0.nc | SSP5 | 4.5 Wm^−2^ | NO_X_ |
| AIM-SSPRCP-Emission-SSP5-45-OCE-v.1.0.nc | SSP5 | 4.5 Wm^−2^ | OC |
| AIM-SSPRCP-Emission-SSP5-45-SO2-v.1.0.nc | SSP5 | 4.5 Wm^−2^ | Sulphur |
| AIM-SSPRCP-Emission-SSP5-45-VOC-v.1.0.nc | SSP5 | 4.5 Wm^−2^ | VOC |
| AIM-SSPRCP-Emission-SSP5-60-BCE-v.1.0.nc | SSP5 | 6.0 Wm^−2^ | BC |
| AIM-SSPRCP-Emission-SSP5-60-CH4-v.1.0.nc | SSP5 | 6.0 Wm^−2^ | CH_4_ |
| AIM-SSPRCP-Emission-SSP5-60-COE-v.1.0.nc | SSP5 | 6.0 Wm^−2^ | CO |
| AIM-SSPRCP-Emission-SSP5-60-NH3-v.1.0.nc | SSP5 | 6.0 Wm^−2^ | NH_3_ |
| AIM-SSPRCP-Emission-SSP5-60-NOX-v.1.0.nc | SSP5 | 6.0 Wm^−2^ | NO_X_ |
| AIM-SSPRCP-Emission-SSP5-60-OCE-v.1.0.nc | SSP5 | 6.0 Wm^−2^ | OC |
| AIM-SSPRCP-Emission-SSP5-60-SO2-v.1.0.nc | SSP5 | 6.0 Wm^−2^ | Sulphur |
| AIM-SSPRCP-Emission-SSP5-60-VOC-v.1.0.nc | SSP5 | 6.0 Wm^−2^ | VOC |
| AIM-SSPRCP-Emission-SSP5-Baseline-BCE-v.1.0.nc | SSP5 | Baseline | BC |
| AIM-SSPRCP-Emission-SSP5-Baseline-CH4-v.1.0.nc | SSP5 | Baseline | CH_4_ |
| AIM-SSPRCP-Emission-SSP5-Baseline-COE-v.1.0.nc | SSP5 | Baseline | CO |
| AIM-SSPRCP-Emission-SSP5-Baseline-NH3-v.1.0.nc | SSP5 | Baseline | NH_3_ |
| AIM-SSPRCP-Emission-SSP5-Baseline-NOX-v.1.0.nc | SSP5 | Baseline | NO_X_ |
| AIM-SSPRCP-Emission-SSP5-Baseline-OCE-v.1.0.nc | SSP5 | Baseline | OC |
| AIM-SSPRCP-Emission-SSP5-Baseline-SO2-v.1.0.nc | SSP5 | Baseline | Sulphur |
| AIM-SSPRCP-Emission-SSP5-Baseline-VOC-v.1.0.nc | SSP5 | Baseline | VOC |

Supplementary Table S 3: Full list of land-use files

| File name | Socioeconomic assumption | Climate mitigation level |
| --- | --- | --- |
| AIM-SSPRCP-LUMap-SSP1_19-v.1.0-v.1.0.nc | SSP1 | 1.9 Wm^−2^ |
| AIM-SSPRCP-LUMap-SSP1_26-v.1.0-v.1.0.nc | SSP1 | 2.6 Wm^−2^ |
| AIM-SSPRCP-LUMap-SSP1_34-v.1.0-v.1.0.nc | SSP1 | 3.4 Wm^−2^ |
| AIM-SSPRCP-LUMap-SSP1_45-v.1.0-v.1.0.nc | SSP1 | 4.5 Wm^−2^ |
| AIM-SSPRCP-LUMap-SSP1_Baseline-v.1.0-v.1.0.nc | SSP1 | Baseline |
| AIM-SSPRCP-LUMap-SSP2_19-v.1.0-v.1.0.nc | SSP2 | 1.9 Wm^−2^ |
| AIM-SSPRCP-LUMap-SSP2_26-v.1.0-v.1.0.nc | SSP2 | 2.6 Wm^−2^ |
| AIM-SSPRCP-LUMap-SSP2_34-v.1.0-v.1.0.nc | SSP2 | 3.4 Wm^−2^ |
| AIM-SSPRCP-LUMap-SSP2_45-v.1.0-v.1.0.nc | SSP2 | 4.5 Wm^−2^ |
| AIM-SSPRCP-LUMap-SSP2_60-v.1.0-v.1.0.nc | SSP2 | 6.0 Wm^−2^ |
| AIM-SSPRCP-LUMap-SSP2_Baseline-v.1.0-v.1.0.nc | SSP2 | Baseline |
| AIM-SSPRCP-LUMap-SSP3_34-v.1.0-v.1.0.nc | SSP1 | 3.4 Wm^−2^ |
| AIM-SSPRCP-LUMap-SSP3_45-v.1.0-v.1.0.nc | SSP1 | 4.5 Wm^−2^ |
| AIM-SSPRCP-LUMap-SSP3_60-v.1.0-v.1.0.nc | SSP3 | 6.0 Wm^−2^ |
| AIM-SSPRCP-LUMap-SSP3_Baseline-v.1.0-v.1.0.nc | SSP3 | Baseline |
| AIM-SSPRCP-LUMap-SSP4_26-v.1.0-v.1.0.nc | SSP4 | 2.6 Wm^−2^ |
| AIM-SSPRCP-LUMap-SSP4_34-v.1.0-v.1.0.nc | SSP4 | 3.4 Wm^−2^ |
| AIM-SSPRCP-LUMap-SSP4_45-v.1.0-v.1.0.nc | SSP4 | 4.5 Wm^−2^ |
| AIM-SSPRCP-LUMap-SSP4_Baseline-v.1.0-v.1.0.nc | SSP4 | Baseline |
| AIM-SSPRCP-LUMap-SSP5_26-v.1.0-v.1.0.nc | SSP5 | 2.6 Wm^−2^ |
| AIM-SSPRCP-LUMap-SSP5_34-v.1.0-v.1.0.nc | SSP5 | 3.4 Wm^−2^ |
| AIM-SSPRCP-LUMap-SSP5_45-v.1.0-v.1.0.nc | SSP5 | 4.5 Wm^−2^ |
| AIM-SSPRCP-LUMap-SSP5_60-v.1.0-v.1.0.nc | SSP5 | 6.0 Wm^−2^ |
| AIM-SSPRCP-LUMap-SSP5_Baseline-v.1.0-v.1.0.nc | SSP5 | Baseline |

Supplementary Figure S 1: Geographic regions in the AIM/CGE model. See Table 2 for the definitions of the regional codes.


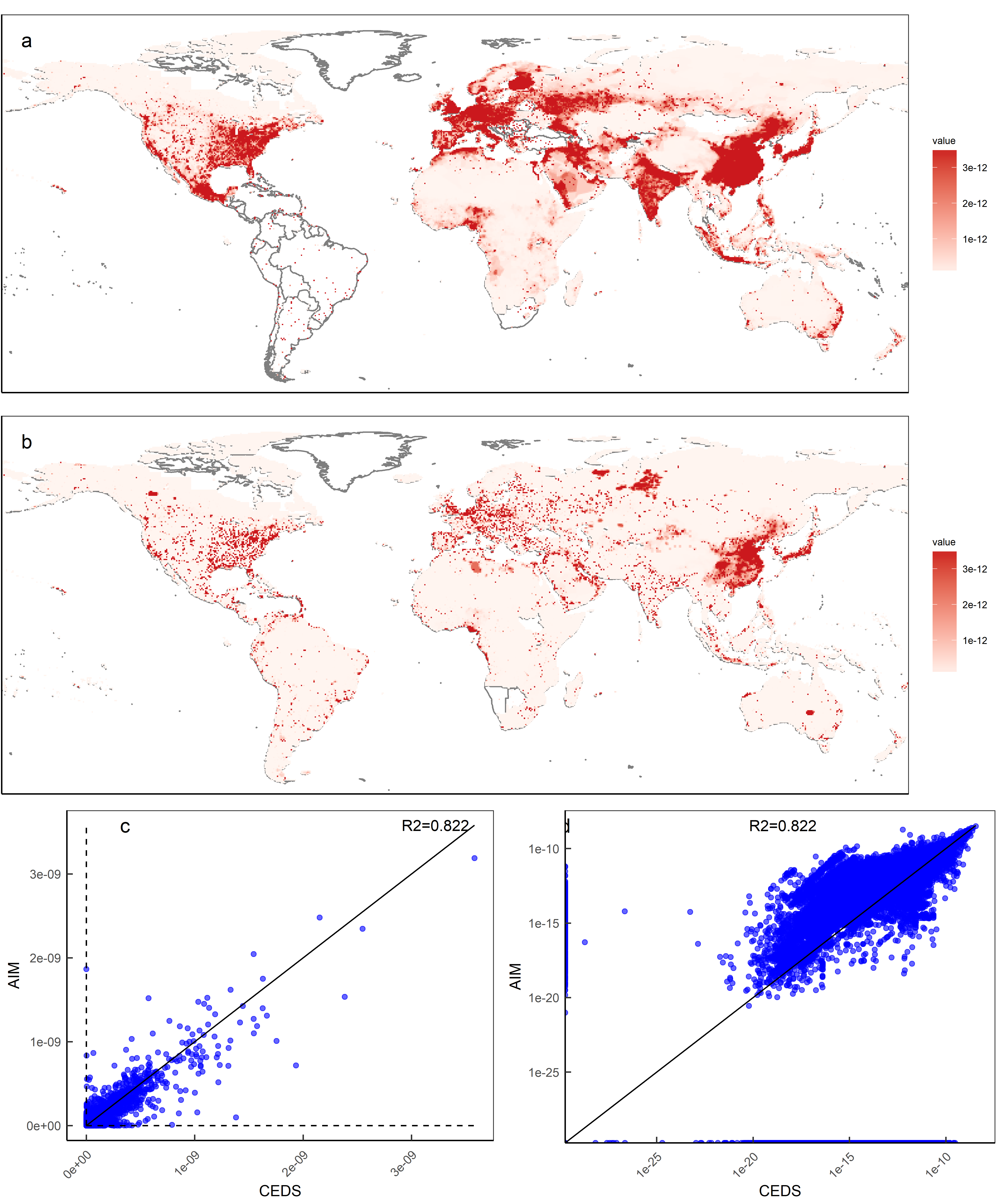


Supplementary Figure S 2: Comparison of downscaled NO_X_ emissions from the energy sector in 2010.

(a) Spatial emission density for AIM-SSP/RCP. (b) Spatial emission density for CEDS. (c) the datasets on normal scales. (d) The datasets on logarithmic scales. All panels use the same unit (kg/ NO_2_/m^2^).


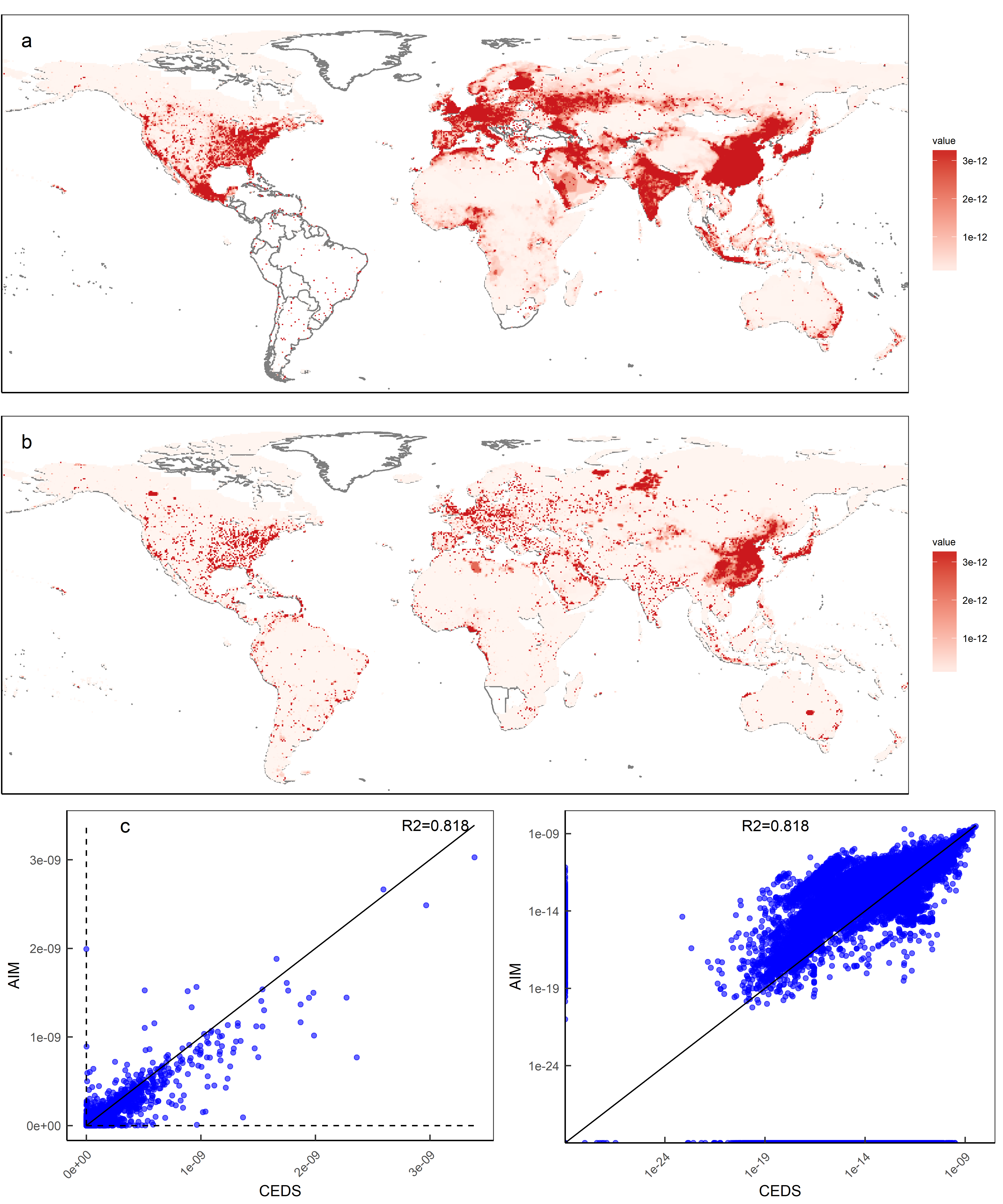


Supplementary Figure S 3: Comparison of the downscaled NO_X_ emissions energy sector in 2010.

(a) Spatial emission density for AIM-SSP/RCP. (b) Spatial emission density for CEDS. (c) the datasets on normal scales. (d) The datasets on logarithmic scales. All panels use the same unit (kg/NO_2_/m^2^).


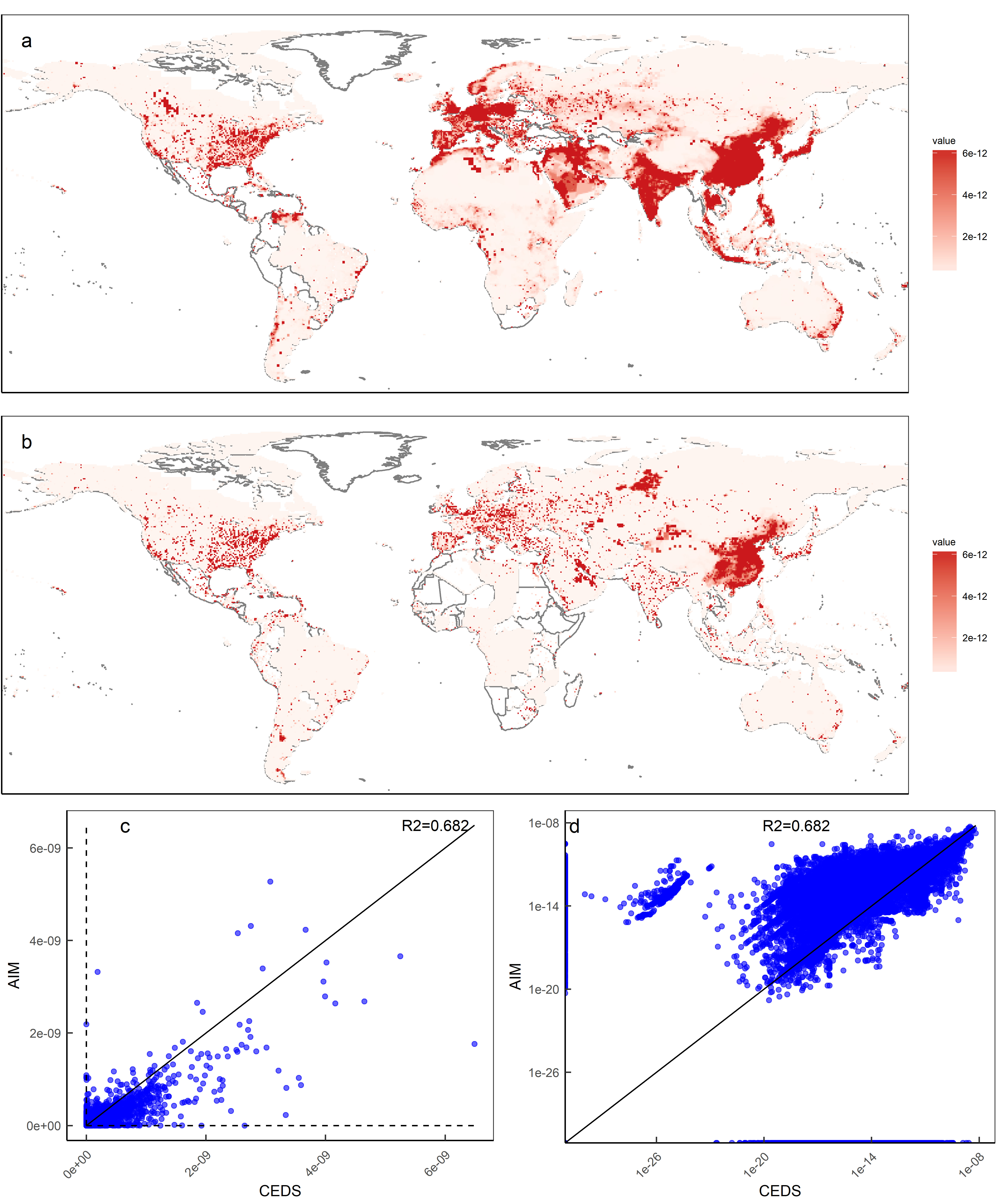


Supplementary Figure S 4: Comparison of the downscaled SO_2_ emissions energy sector in 2010.

(a) Spatial emission density for AIM-SSP/RCP. (b) Spatial emission density for CEDS. (c) the datasets on normal scales. (d) The datasets on logarithmic scales. All panels use the same unit (kg/s/m^2^).


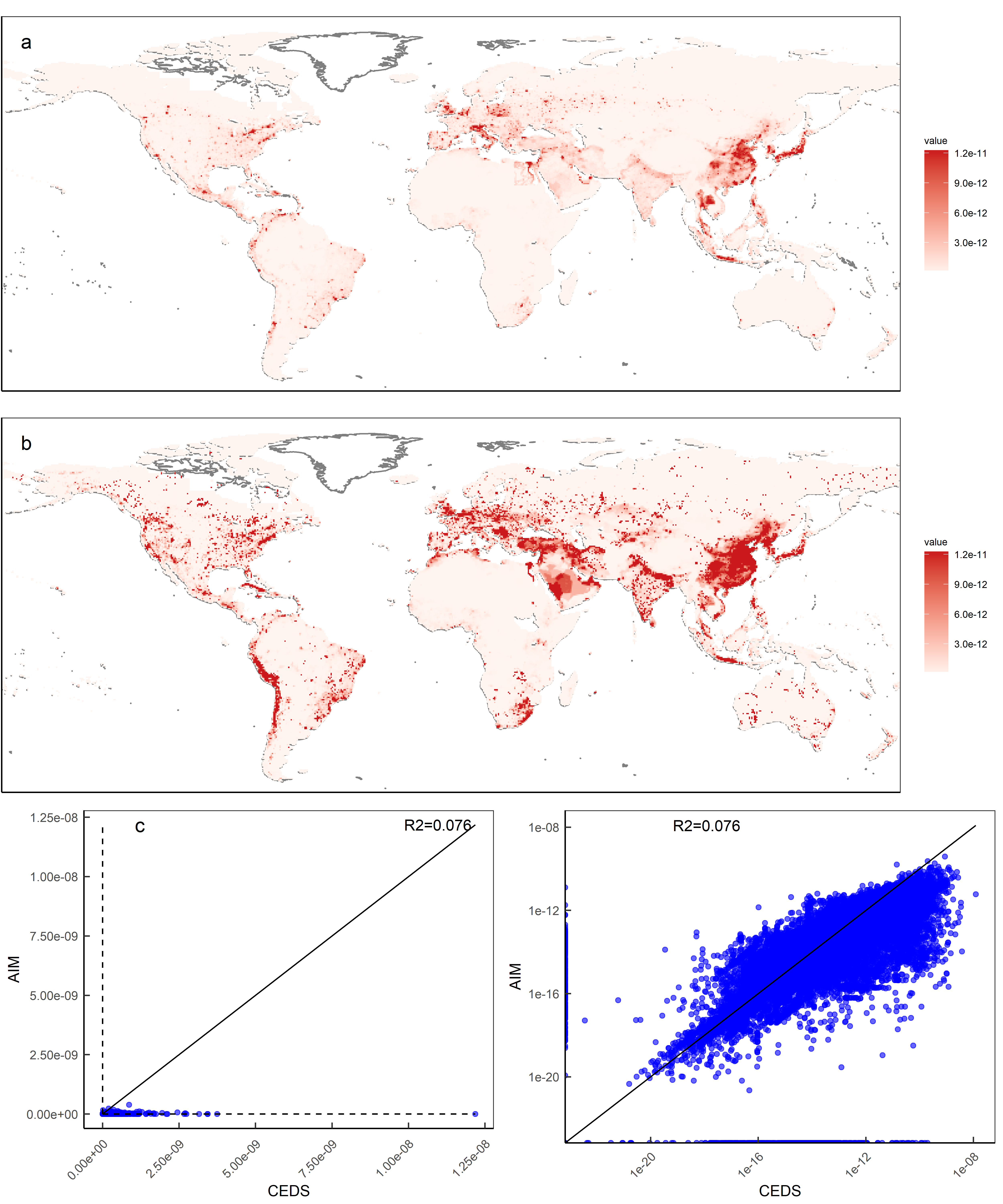


Supplementary Figure S 5: Comparison of the downscaled SO_2_ emissions from the transport sector in 2005.

(a) Spatial emission density for AIM-SSP/RCP. (b) Spatial emission density for CEDS. (c) the datasets on normal scales. (d) The datasets on logarithmic scales. All panels use the same unit (kg/s/m^2^).


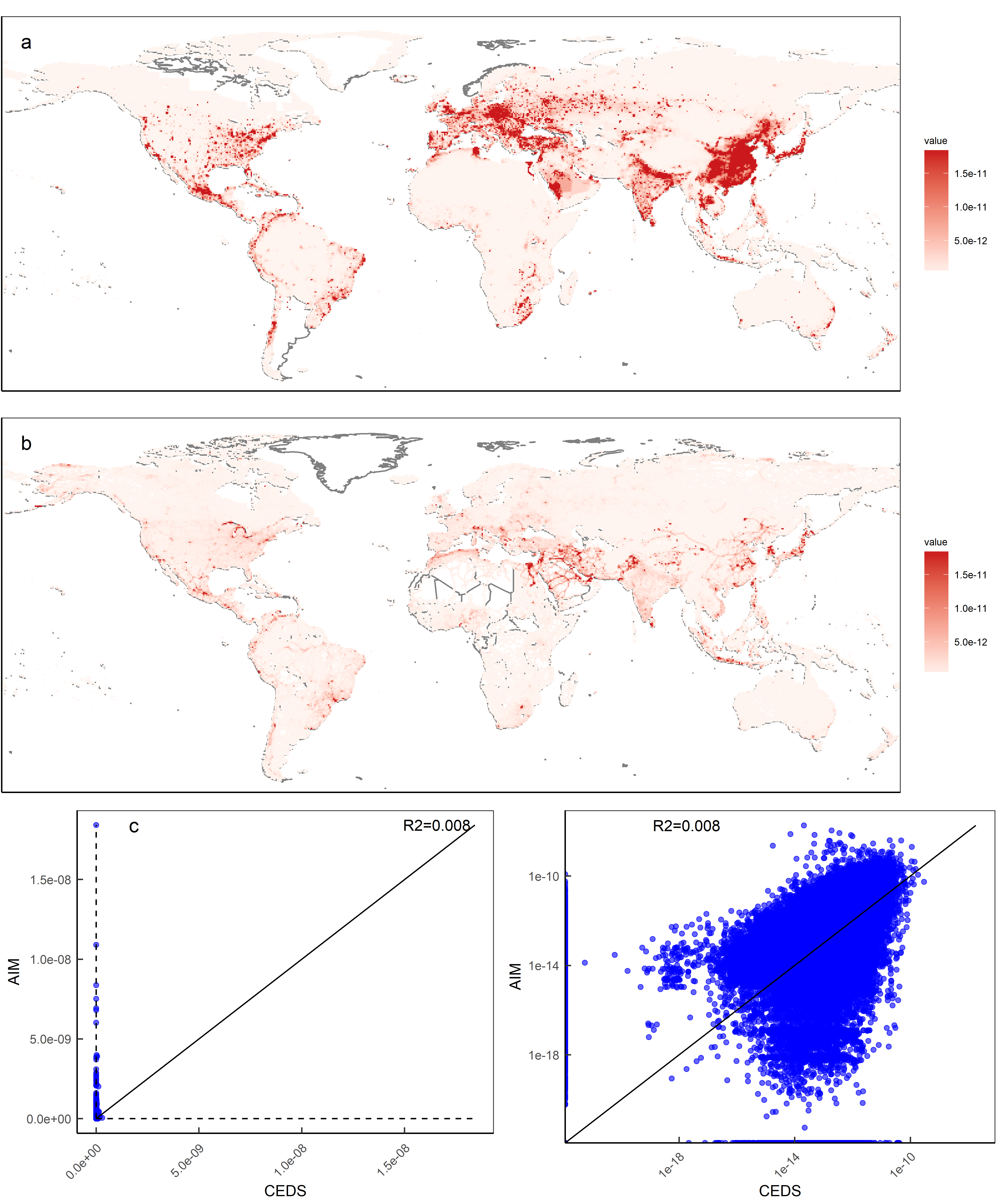


Supplementary Figure S 6: Comparison of the downscaled SO_2_ emissions from the residential and commercial sector in 2005.

(a) Spatial emission density for AIM-SSP/RCP. (b) Spatial emission density for CEDS. (c) the datasets on normal scales. (d) The datasets on logarithmic scales. All panels use the same unit (kg/s/m^2^).


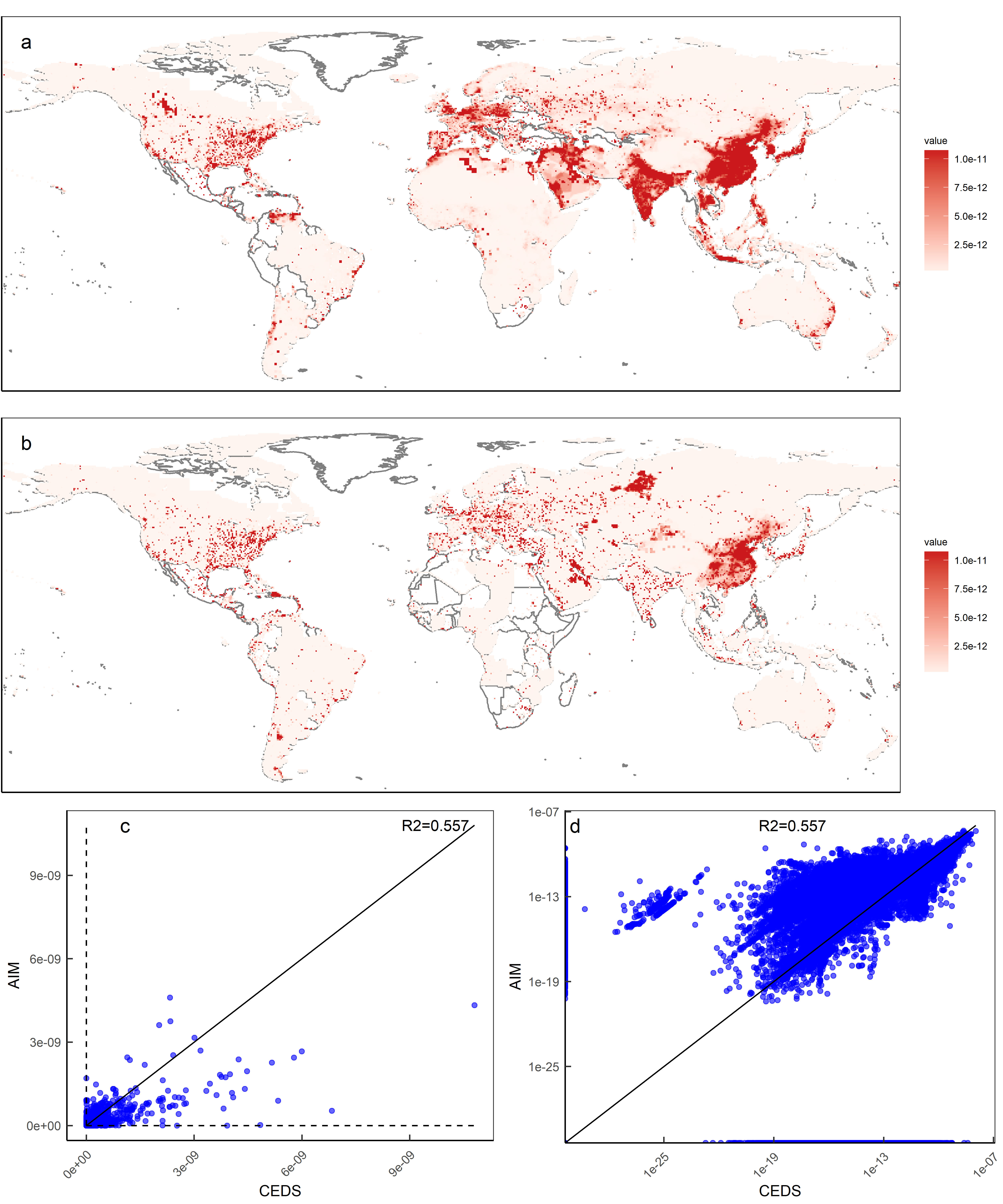


Supplementary Figure S 7: Comparison of the downscaled SO_2_ emissions from the energy sector in 2015.

(a) Spatial emission density for AIM-SSP/RCP. (b) Spatial emission density for ScenarMIP data. (c) the datasets on normal scales. (d) The datasets on logarithmic scales. All panels use the same unit (kg/s/m^2^).


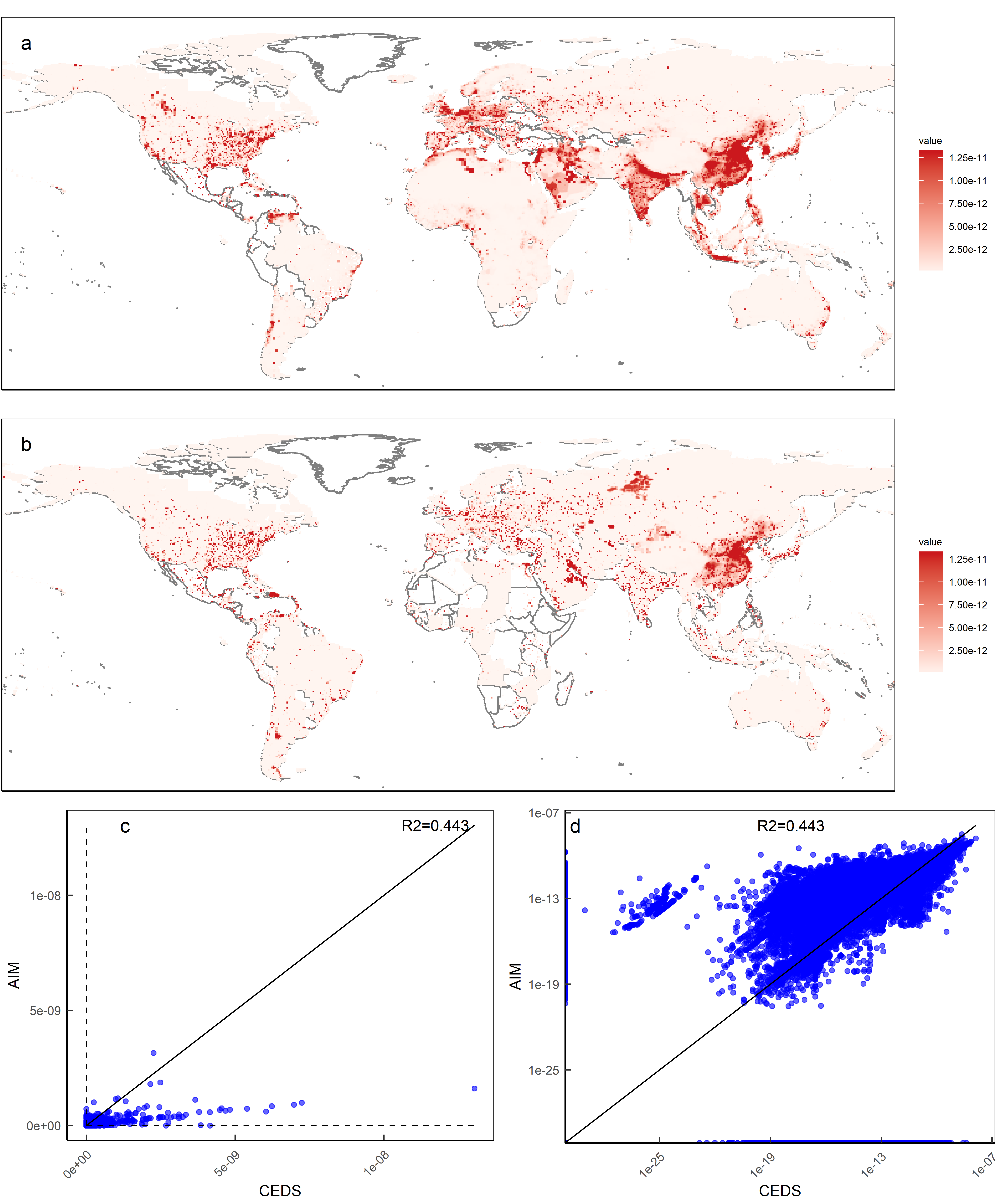


Supplementary Figure S 8: Comparison of the downscaled SO_2_ emissions from the energy sector in 2050.

(a) Spatial emission density for AIM-SSP/RCP. (b) Spatial emission density for ScenarMIP data. (c) the datasets on normal scales. (d) The datasets on logarithmic scales. All panels use the same unit (kg/s/m^2^).


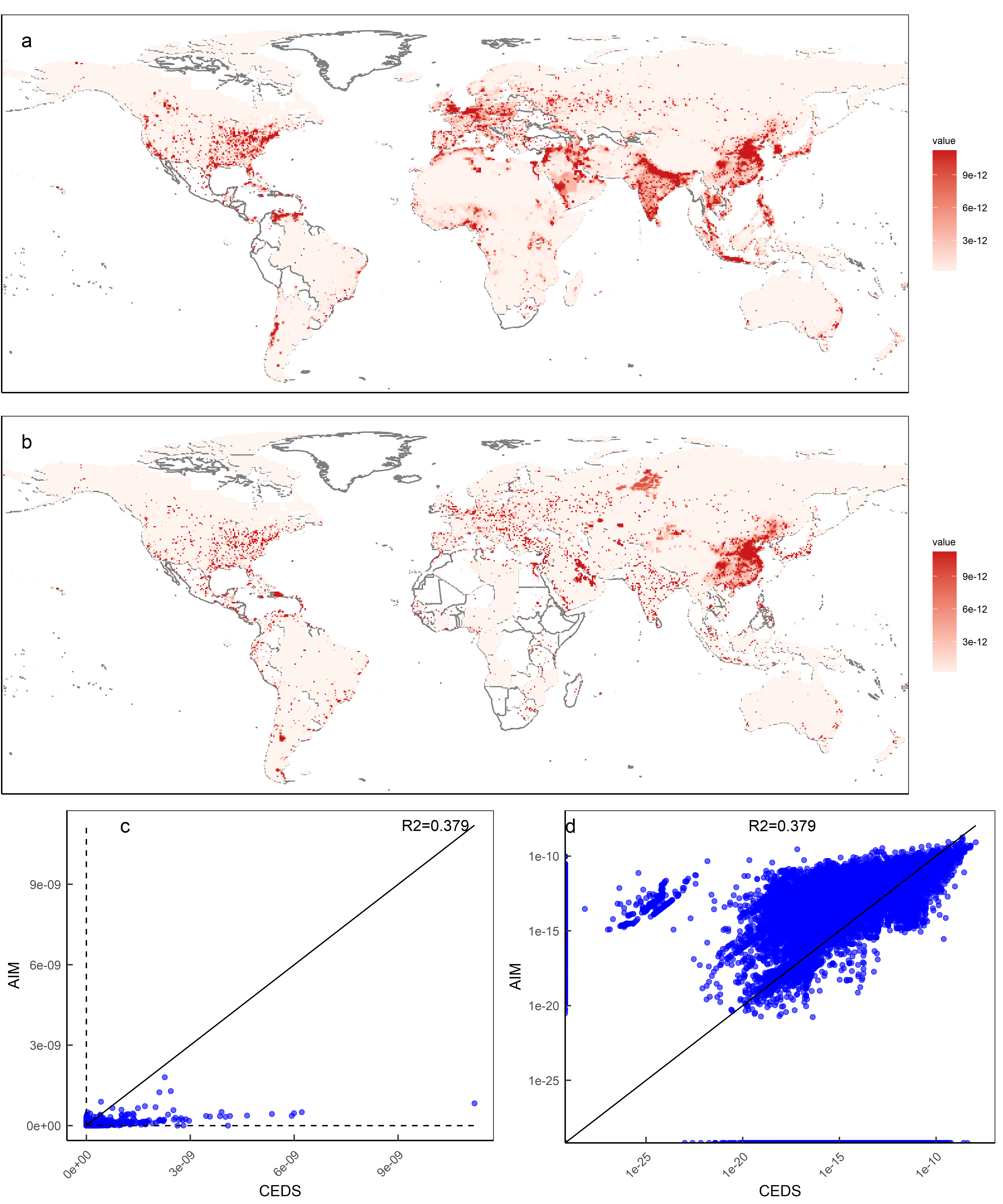


Supplementary Figure S 9: Comparison of the downscaled SO_2_ emissions from the energy sector in 2100.

(a) Spatial emission density for AIM-SSP/RCP. (b) Spatial emission density for ScenarMIP data. (c) the datasets on normal scales. (d) The datasets on logarithmic scales. All panels use the same unit (kg/s/m^2^).


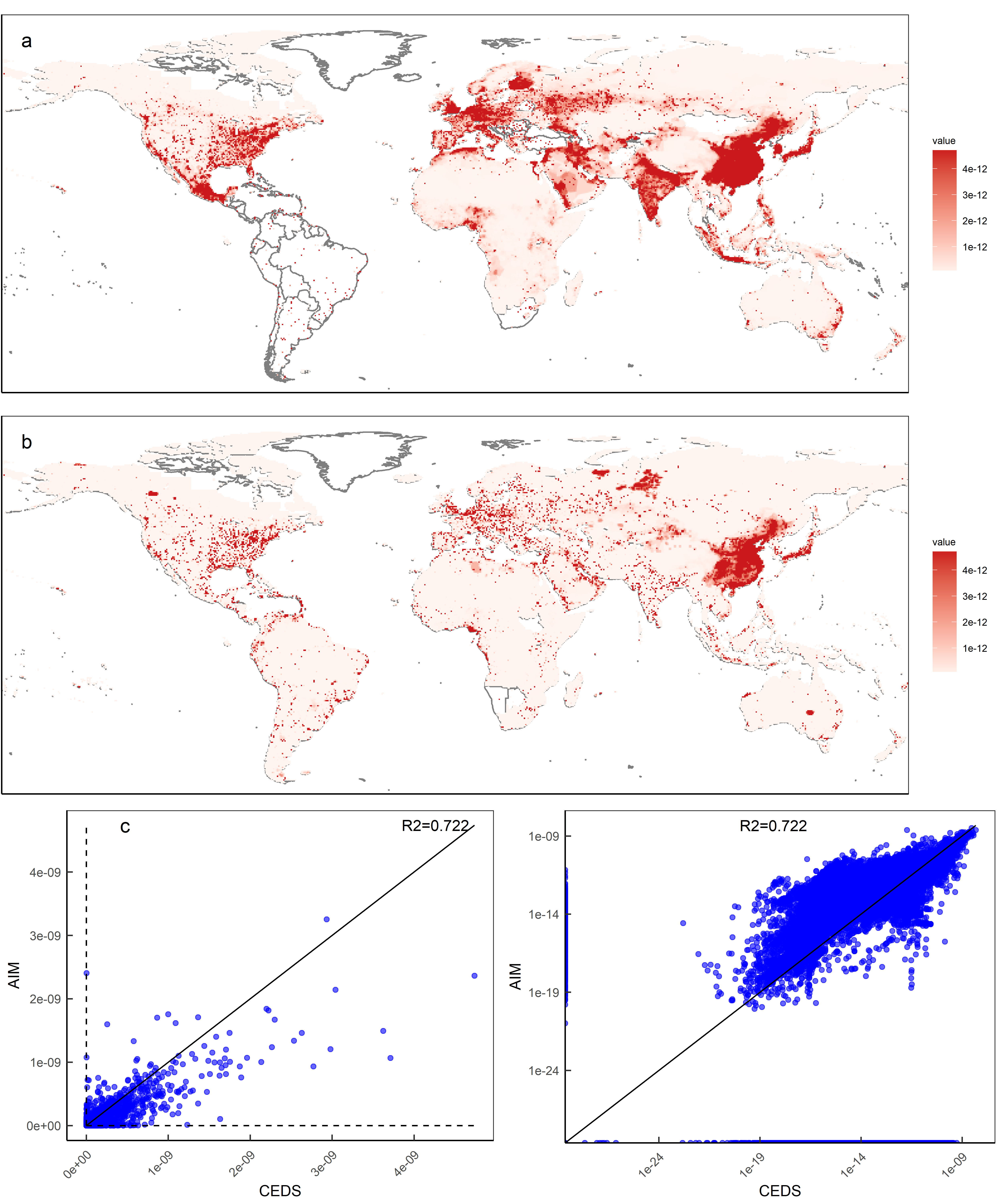


Supplementary Figure S 10: Comparison of the downscaled NOx emissions from the energy sector in 2015.

(a) Spatial emission density for AIM-SSP/RCP. (b) Spatial emission density for ScenarMIP data. (c) the datasets on normal scales. (d) The datasets on logarithmic scales. All panels use the same unit (kg/NO_2_/m^2^).


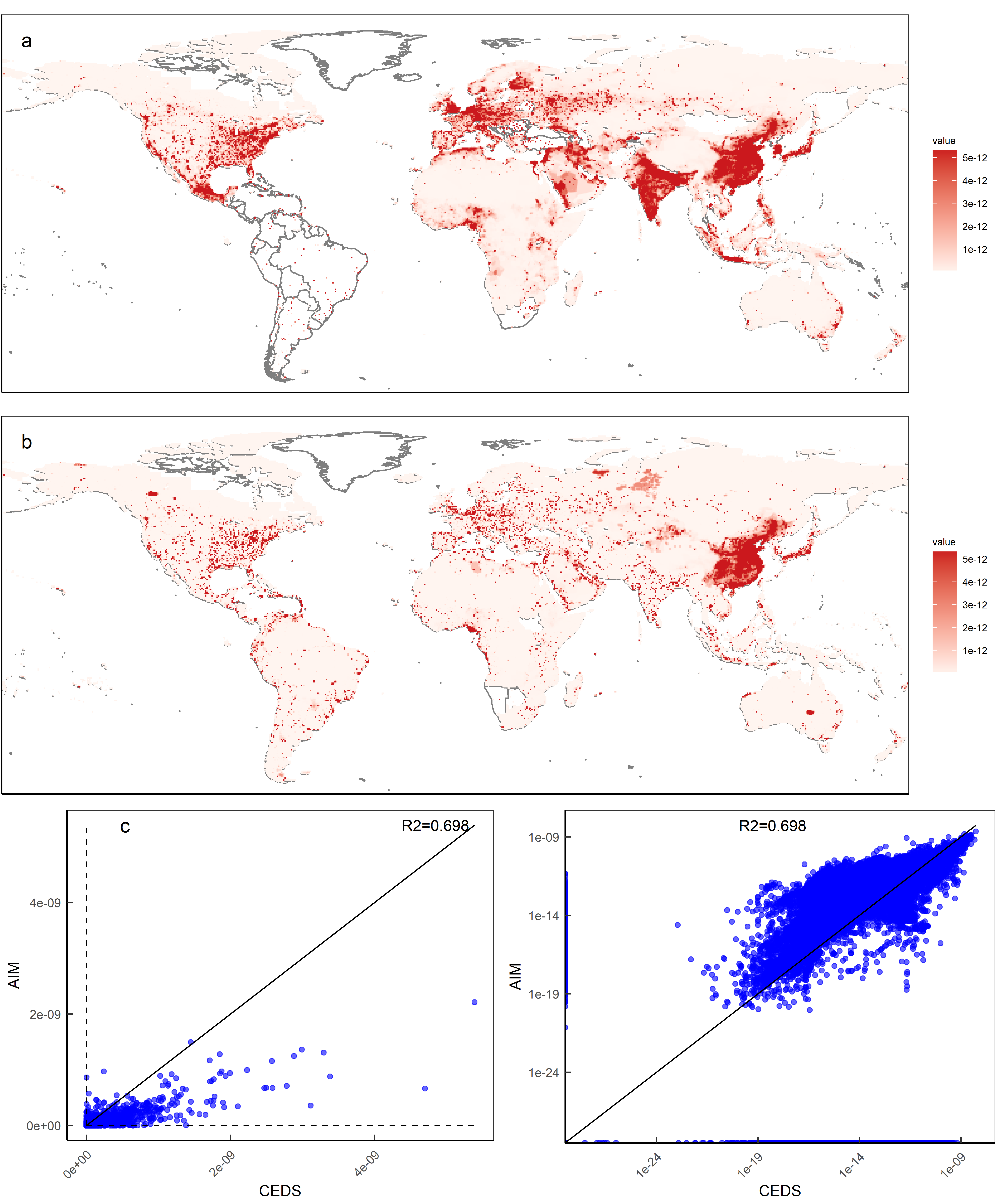


Supplementary Figure S 11: Comparison of the downscaled NOx emissions from the energy sector in 2050.

(a) Spatial emission density for AIM-SSP/RCP. (b) Spatial emission density for ScenarMIP data. (c) the datasets on normal scales. (d) The datasets on logarithmic scales. All panels use the same unit (kg/NO_2_/m^2^).


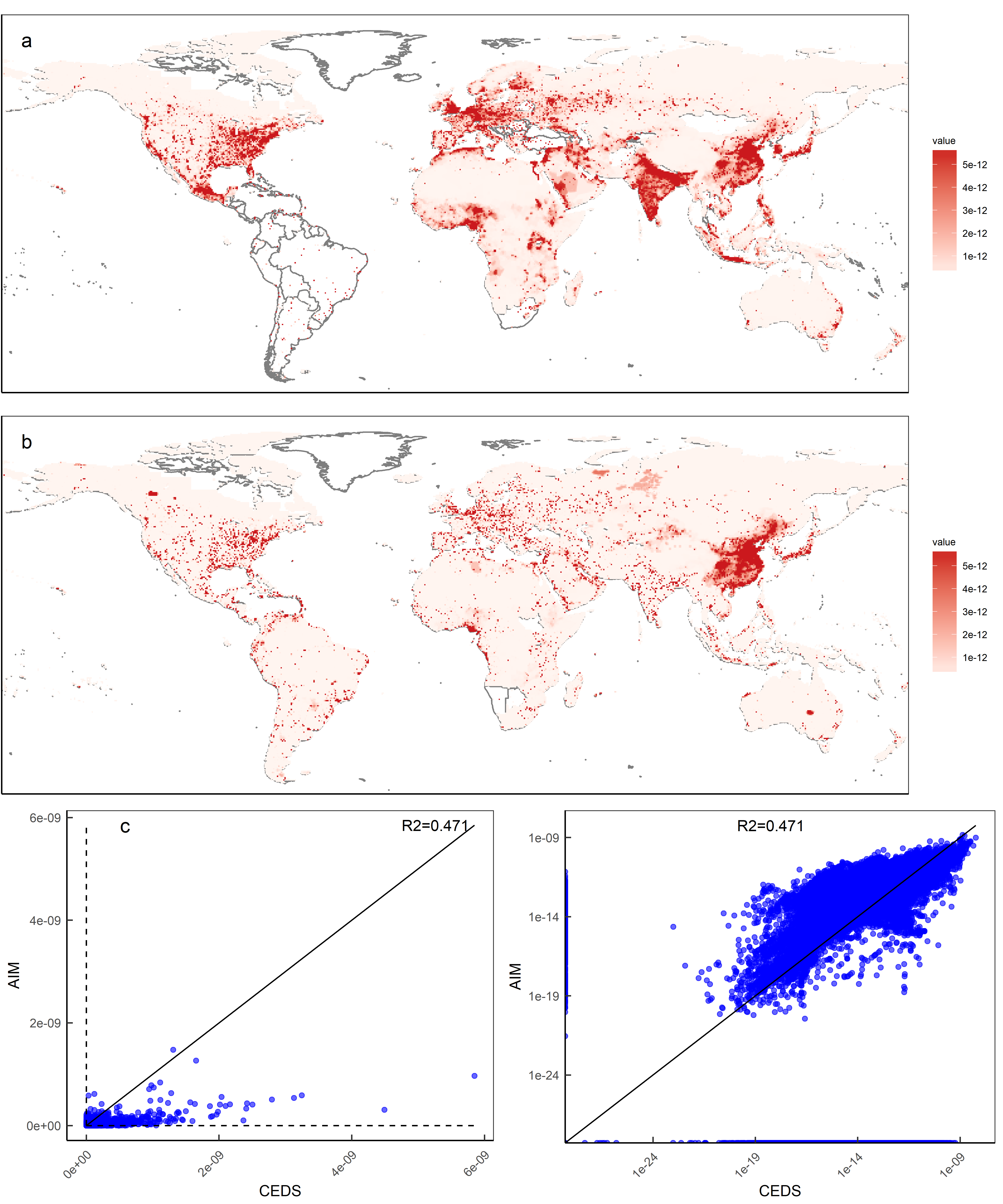


Supplementary Figure S 12: Comparison of the downscaled NOx emissions from the energy sector in 2100.

(a) Spatial emission density for AIM-SSP/RCP. (b) Spatial emission density for ScenarMIP data. (c) the datasets on normal scales. (d) The datasets on logarithmic scales. All panels use the same unit (kg/NO_2_/m^2^).
